# Distribution, host origin, and transmission cycles of *Trypanosoma cruzi* genotypes in the State of Rio Grande do Norte, Brazil

**DOI:** 10.1590/0037-8682-0063-2025

**Published:** 2025-08-08

**Authors:** George Harisson Felinto Sampaio, Nathan Ravi Medeiros Honorato, Lucas Abrantes Batista, Maricélia de Aquino Santana, Carlos Ramon do Nascimento Brito, Rand Randall Martins, Paulo Marcos da Matta Guedes, Andressa Noronha Barbosa da Silva, Lúcia Maria da Cunha Galvão

**Affiliations:** 1 Universidade Federal do Rio Grande do Norte, Programa de Pós-Graduação em Ciências da Saúde, Natal, RN, Brasil.; 2 Universidade Federal de Minas Gerais, Programa de Pós-Graduação em Parasitologia, Belo Horizonte, MG, Brasil.; 3 Universidade Federal do Rio Grande do Norte, Programa de Pós-Graduação em Biologia Parasitária, Natal, RN, Brasil.; 4 Universidade Federal do Rio Grande do Norte, Programa de Pós-Graduação em Ciências Farmacêuticas, Natal, RN, Brasil.

**Keywords:** Trypanosoma cruzi, Genetic diversity, Transmission cycles, Invertebrate and vertebrate hosts, Public health

## Abstract

**Background:**

The genetic diversity of *Trypanosoma cruzi* and the epidemiological characteristics of the environments in which the parasite occurs are essential for understanding infection dynamics and controlling Chagas disease. This systematic review aimed to: (i) identify and analyze studies that evaluated the genetic variability and epidemiological aspects of *T. cruzi* infection in the state of Rio Grande do Norte, Brazil; (ii) summarize the information reported in the literature; and (iii) suggest new control strategies tailored to the region’s epidemiological profile.

**Methods:**

Following the application of inclusion and exclusion criteria, 13 studies catalogued in *PubMed*, the *Brazilian Virtual* Health *Library*, *Scopus*, and the *Web of Science* were selected.

**Results:**

*T. cruzi* isolates were primarily obtained from triatomine species *Triatoma brasiliensis*, *Panstrongylus lutzi*, and *Triatoma pseudomaculata*, as well as from wild mammals such as *Euphractus sexcinctus*, *Galea spixii*, and humans. A total of 295 *T. cruzi* isolates were genotyped: 46.5% (137) were identified as Discrete Typing Unit (DTU) I, 29.1% (86) as DTU II, and 20% (59) as DTU III. Mixed infections were detected in 4.4% (13/295) of hosts. Triatomine species were found in both peridomestic and intradomestic environments and were occasionally infected with *T. cruzi*.

**Conclusions:**

This review provides a comprehensive overview of the circulation of distinct *Trypanosoma cruzi* genotypes (I, II, and III) in both wild and human-modified environments in Rio Grande do Norte.

## INTRODUCTION

Chagas disease (CD), caused by the protozoan parasite *Trypanosoma cruzi* (Chagas, 1909), originated millions of years ago as an enzootic infection among wild nonhuman animals and began to be transmitted as an anthropozoonosis when humans encroached upon sylvatic ecotopes^1^. The parasite is found across mammalian fauna in the Americas and in triatomine insects, ranging from the southern United States to southern Argentina. Two decades ago, CD was thought to be under control in Brazil and no longer posed a significant public health threat. However, the disease has persisted and has re-emerged as a health concern under varying epidemiological scenarios, both within and beyond the Americas^2^. Recent data indicate ongoing challenges in diagnosing and effectively treating human infection, alongside underreporting of morbidity and mortality, which means CD continues to represent a significant public health issue in endemic regions^3^.

Biological differences between *T. cruzi* strains isolated from different hosts have been observed, including variations in trypomastigote morphology, parasitemia levels, mortality curves in experimental models, treatment susceptibility, and tissue tropism^4^ ^,^ ^5^. Biological, biochemical, and molecular studies have demonstrated that *T. cruzi* is a highly heterogeneous taxon. Over time, its population structure has been studied using a variety of research tools and methodologies^6^ ^-^ ^10^. In 2009, a committee of experts proposed an intraspecific nomenclature system recognizing six Discrete Typing Units (DTUs), designated TcI to TcVI, based on data from biological, genetic, and biochemical markers^11^. A seventh DTU, Tcbat, was later identified in several bat species in Brazil^12^. More recent phylogenetic and phylogeographic studies using multiple molecular markers specific to Tcbat have definitively supported its classification as a distinct DTU^13^.

Consequently, investigating the intraspecific genetic variability of *Trypanosoma cruzi* is essential for better understanding its relationship with different environments and transmission cycles. The diversity of triatomine vectors and mammalian hosts likely plays a significant role in the distribution of different DTUs and may help maintain *T. cruzi* heterogeneity by facilitating the emergence of new variants over time through natural selection and genetic drift^14^.

The Northeast region of Brazil holds considerable epidemiological importance in the context of Chagas disease due to its high vulnerability to the chronic form of the disease and its ranking as the region with the second -highest incidence in the country^15^. The Caatinga biome dominates much of this region, and *Triatoma brasiliensis* (Neiva, 1911) is the primary vector. This species is currently found in both sylvatic and anthropogenic environments and has adapted to various ecotopes in peridomestic and intradomestic settings^15^ ^,^ ^16^. Furthermore, its broad mammalian host preference for blood meals increases the chances of repeated infections with different DTUs, thereby raising the likelihood of mixed infections within individual vectors^16^ ^-^ ^18^.

Given this context, it is crucial to identify the *T. cruzi* genotypes circulating in the different mesoregions of Rio Grande do Norte (RN), an administrative state located in northeastern Brazil, to improve our understanding of local transmission dynamics. Accordingly, a systematic review was conducted to identify and analyze studies that evaluated the genetic variability and epidemiological aspects of *T. cruzi* infection in the region, and to compile existing literature that could inform the development of new, targeted control strategies.

## METHODS

### Search strategy and eligibility criteria

This systematic review was conducted following the Preferred Reporting Items for Systematic Reviews and Meta-Analyses Protocols (PRISMA-P) guidelines^19^ and was registered in the PROSPERO database (ID: 403597). Searches were performed in *PubMed*, the *Brazilian Virtual* Health *Library (BVS*), and *Scopus*. The search strategy was adapted for each database using the following terms and Boolean operators: for *PubMed* - (*Trypanosoma cruzi*) AND (*Rio Grande do Norte* State); for *BVS* - (*Trypanosoma cruzi*) AND (*Rio Grande do Norte* State); and for *Scopus* - *Trypanosoma cruzi* AND *Rio Grande do Norte* State. We searched for articles published between January 2010 and January 2025. Searches were carried out between January 2024 and January 2025.

Two authors (NRMH and ANBS) independently screened all retrieved records based on titles and abstracts using the following eligibility criteria: studies involving genetic characterization of *T. cruzi* isolates obtained from either vertebrate or invertebrate hosts in the state of Rio Grande do Norte, Brazil. All types of primary research were included, including clinical, preclinical, and observational studies. However, secondary literature (e.g., reviews) and gray literature (e.g., theses, dissertations, and course- conclusion papers) were excluded. No language restrictions were applied. When necessary, a third author (ANBS) resolved disagreements regarding study inclusion. 

### Data extraction

**FIGURE 1:**
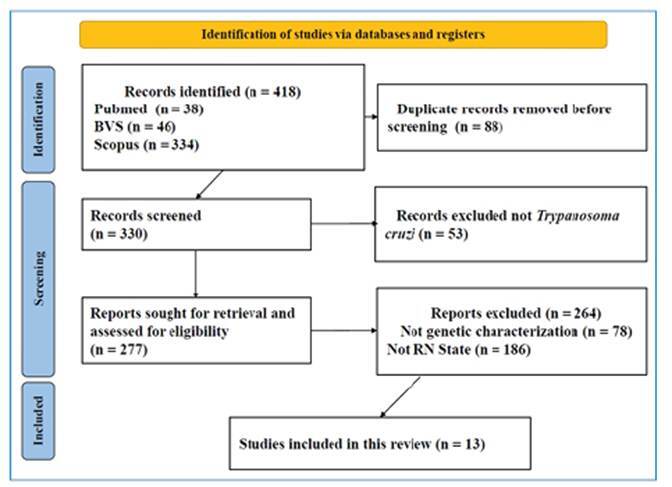
Identification of studies via databases and registries.

Following the initial screening, the full texts of the selected articles were independently reviewed to confirm eligibility. Some articles were excluded at this stage because the data they contained did not meet the selection criteria. The detailed screening and selection process is illustrated in the PRISMA flowchart (Figure 1).

From the final set of included articles, the following data were extracted: year of publication, authors, *T. cruzi* isolate name, DTU type, DNA extraction and characterization method, host species from which the sample was obtained, transmission cycle associated with the isolate, and the municipality where the sample was collected (see Supplementary Material).

## RESULTS

**FIGURE 2:**
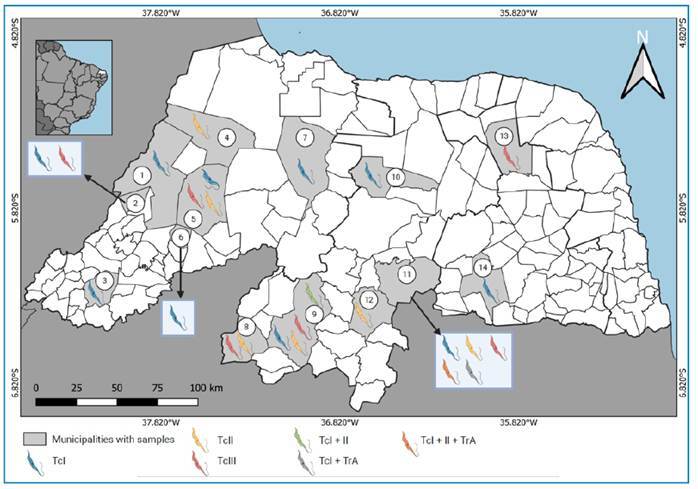
Map of the state of Rio Grande do Norte in northeastern Brazil showing the location and DTU type of the *Trypanosoma cruzi* isolates sampled in the 13 previous studies included in the systematic review. The municipalities in which *T. cruzi*-positive samples were found are shaded in light gray, while the different colors of the *T. cruzi* symbols indicate the different DTU types(s), as explained in the legend.

The eligibility criteria were first applied to the titles and abstracts of documents retrieved from public databases and then to the full texts of a subset of these initially selected articles. A total of 418 articles were identified and assessed for eligibility. Following full-text screening, 88 articles were excluded due to duplicate data, 53 for not referring to *T. cruzi*, 78 for not reporting parasite genotyping, and 186 because the geographical origin of the isolates was not within the state of the RN. Ultimately, 13 articles met all criteria and were included in this systematic review (see Supplementary Material). These studies identified *T. cruzi* isolates from various municipalities across RN, as shown in Figure 2.

The geographical distribution and genotypes of *T. cruzi* isolates reported in the 13 included studies, each conducted in different municipalities of RN, are summarized in Figure 2. In total, 295 *T. cruzi* isolates were identified across the studies: 46.4% (137) were genetically characterized as DTU I, 29.2% (86) as DTU II, and 20% (59) as DTU III. Mixed infections were also detected: DTU I + II in 3.4% (10) of cases, DTU I + *Trypanosoma rangeli* (Tejera, 1920) genotype A (TrA) in 0.7% (2), and DTU I + II + TrA in 0.3% (1).

DTU I isolates were identified in 2.7% of the *Homo sapiens sapiens* (Linnaeus, 1758) samples (n = 8) and in three triatomine species: *Triatoma brasiliensis* (43.1%; n = 127), *Triatoma pseudomaculata* (Corrêa & Espínola, 1964) (0.3%; n = 1), and *Triatoma petrochiae* (Pinto, 1927) (0.3%; n = 1). Among the 86 isolates characterized as DTU II, 19.7% (n = 58) were obtained from *T. brasiliensis*, 9.2% (n = 27) from *H. sapiens*, and 0.3% (n = 1) from *Ovis aries* (Linnaeus, 1758). *Trypanosoma cruzi* DTU III isolates were derived from five different species: 9.8% (n = 29) from *Panstrongylus lutzi* (Neiva & Pinto, 1923), 7.1% (n = 21) from *T. brasiliensis*, 1.7% (n = 5) from *H. sapiens*, 1.1% (n = 3) from *Euphractus sexcinctus* (Linnaeus, 1758), and 0.3% (n = 1) from *Galea spixii* (Wagler, 1831) (Table 1).

**TABLE 1:** Genotyping and hosts of the *Trypanosoma cruzi* isolates sampled in the 13 previous studies included in the systematic review in the state of Rio Grande do Norte, Brazil.

	Percentage (and number) of Discrete Typing Units (DTUs)						
Host							I	II	III	I+II	I+TrA	I+II+TrA	Total
*Triatoma brasiliensis*	43.1 (127)	19.7 (58)	7.1 (21)	3.1 (9)	0.7 (2)	0.3 (1)	**74.0 (218)**
*Homo sapiens sapiens*	2.7 (8)	9.2 (27)	1.7 (5)	-	-	-	**13.6 (40)**
*Panstrongylus lutzi*	-	-	9.8 (29)	0.3 (1)	-	-	**10.1 (30)**
*Euphractus sexcinctus*	-	-	1.1 (3)	-	-	-	**1.1 (3)**
*Triatoma pseudomaculata*	0.3 (1)	-	-	-	-	-	**0.3 (1)**
*Triatoma petrocchiae*	0.3 (1)	-	-	-	-	-	**0.3 (1)**
*Galea spixii*	-	-	0.3 (1)	-	-	-	**0.3 (1)**
*Ovis aries*	-	0.3 (1)	-	-	-	-	**0.3 (1)**
**Total**	**46.4 (137)**	**29.2 (76)**	**20.0 (59)**	**3.4 (10)**	**0.7 (2)**	**0.3 (1)**	**100.0 (295)**

The isolates reported and genotyped in the 13 studies included in this systematic review were primarily collected from peridomestic environments (44.2 %; n = 131). Of the total isolates analyzed, 33.5% (n = 99) were classified as DTU I, 4.4% (n = 13) as DTU II, and 4.0% (n = 12) as DTU III. Mixed infections were also identified, with DTUs I + II present in 1.7 % (n = 5) of samples, DTU I + *T. rangeli* genotype A (TrA) in 0.3 % (n = 1), and DTUsI + II + TrA in 0.3 % (n = 1).

The sylvatic environment was the second most common source of genotyped isolates (37.6 %; 111/295). Within this setting, DTU II was the most prevalent genotype, accounting for 15.9% (n = 46) of total isolates, followed by DTU III (12.2 %; n = 36) and DTU I (9.5 %; n = 28). The intradomestic environment contributed 14.9% (n = 44) of the total isolates, primarily consisting of DTU II (9.2%; n = 27), followed by DTU I (3.3%; n = 10) and DTU III (2.4%; n = 7) (Table 2). More detailed results from each of the included studies can be found in the Supplementary Material.

**TABLE 2:** Percentages (and numbers) of *Trypanosoma cruzi* DTUs sampled in different transmission environments by the 13 previous studies included in the systematic review in the state of Rio Grande do Norte, Brazil.

DTU	Intradomicile	Peridomicile	Sylvatic	Undefined	Total
**I**	3.3 (10)	33.5 (99)	9.5 (28)	-	**46.3 (137)**
**II**	9.2 (27)	4.4 (13)	15.9 (46)	-	**29.5 (86)**
**III**	2.4 (7)	4.0 (12)	12.2 (36)	1.3 (4)	**19.9 (59)**
**I+II**	-	1.7 (5)	-	1.7 (5)	**3.4 (10)**
**I+TrA**	-	0.3 (1)	-	0.3 (1)	**0.6 (2)**
**I+II+TrA**	-	0.3 (1)	-	-	**0.3 (1)**
**Total**	**14.9 (44)**	**44.2 (131)**	**37.6 (111)**	**3.3 (10)**	**100.0 (295)**

**DTU:** discrete typing unit; **TrA:** *Trypanosoma rangeli* A.

## DISCUSSION

In this systematic review, we compiled data from 13 previously published studies and summarized information on the genotypes of *Trypanosoma cruzi* isolates identified in the state of Rio Grande do Norte, Brazil. DTUs I, II, and III were detected in both single and mixed infections (the latter referring to the presence of more than one DTU in a single sample), as well as in combination with *Trypanosoma rangeli*. In our study area, *T. cruzi* circulates in both sylvatic and anthropogenic environments and has been identified in four species of triatomine vectors, three species of wild mammals, and in humans. This review of the existing literature highlights overlapping areas of occurrence for different *T. cruzi* genotypes and reveals a complex epidemiological landscape of parasite transmission in this part of northeastern Brazil.

Different *T. cruzi* genotypes are widely distributed across the American continent, with DTU I being the most geographically widespread. In Brazil, DTUs I, II, III, and IV are the most frequently reported, with DTU II most commonly associated with human infections, and the other DTUs primarily linked to sylvatic transmission cycles^20^. However, these transmission cycles are not fully delineated, and their spatial boundaries remain poorly defined. Factors such as the mobility of infected synanthropic animals, capable of moving between environments, and human encroachment into sylvatic areas, which alter natural habitats, contribute to this uncertainty. As a result, co-occurrence of different genotypes in the same areas is common, and mixed infections in both invertebrate and vertebrate hosts are frequently observed, as documented in the state of RN. This study provides evidence that changes in the parasite genome can lead to hybridization events^20^ ^,^ ^21^.

This review demonstrated that DTU I was identified in approximately 50% of *Trypanosoma cruzi* infections recorded in the state of RN, primarily in *Triatoma brasiliensis* and humans, and across both sylvatic and peridomestic environments^17^ ^,^ ^20^ ^-^ ^27^. DTU I exhibits a wide distribution pattern consistent with long-term evolutionary history and is considered the most ancient ancestral genotype^14^ ^,^ ^28^. In a separate literature review analyzing 6,343 *T. cruzi* isolates from 19 countries, DTU I was found to be the most prevalent genotype, accounting for approximately 60% of all isolates. In Brazil, DTU I is also the most frequently reported genotype, followed by DTU II^14^. In the present study, DTU I was similarly the most common, widely distributed, and often co-occurring with DTU II^22^ ^,^ ^25^ and, in some cases, with both DTUs II and III^17^ ^,^ ^23^ ^-^ ^27^.

Human infection with DTU I is prevalent in northern South America, Central America, and Mexico and is often associated with Chagas heart disease. In the Brazilian Amazon, DTU I is the primary cause of acute and cardiac forms of Chagas disease, though it is not typically linked to megasyndromes^28^. Although the number of *T. cruzi* isolates obtained from humans in RN was relatively low, DTU I has been associated with chronic Chagas disease in patients presenting with indeterminate, cardiac, digestive, and cardio-digestive clinical forms^25^.

Our results also showed that DTU I isolates were obtained from *T. brasiliensis*, *T. pseudomaculata*, and *T. petrochiae*, suggesting a potential association between DTU I and members of the genus *Triatoma* (Laporte, 1832). This association may contribute to the sustained transmission of DTU I in the region^12^ ^,^ ^17^ ^,^ ^22^ ^-^ ^24^. Notably, *T. brasiliensis* infected with DTU I was implicated in a 2016 oral outbreak of *T. cruzi* infection in the municipality of Marcelino Vieira (RN), linked to sugarcane juice contamination^29^.

DTU II was the second most common genotype identified in the state of RN, occurring across all three transmission environments -sylvatic, peridomestic, and intradomestic -with the highest frequency observed in sylvatic settings. This genotype was also found in *T. brasiliensis*, Ovis aries, and humans^17^ ^,^ ^22^ ^,^ ^23^ ^,^ ^25^ ^,^ ^27^ ^,^ ^30^ ^-^ ^32^. DTU II has demonstrated low levels of genetic variability across different hosts and geographic locations in semi-arid regions26. In other parts of Brazil, DTU II is more frequently associated with the domestic transmission cycle and has been implicated in numerous human infections presenting with various clinical forms of Chagas disease. This genotype has been identified in patients in the chronic phase of Chagas disease with indeterminate, cardiac, digestive, and cardio-digestive clinical forms, supporting previous findings that DTU II plays a significant role in human disease in Brazil^33^ ^,^ ^34^. In RN, DTU II has been similarly associated with indeterminate, cardiac, and digestive clinical manifestations^25^.

*Trypanosoma cruzi* DTU III was identified in RN in two vector species, *T. brasiliensis* and Panstrongylus *lutzi*, and two wild mammal species, *E. sexcinctus* and *G. spixii*, which together likely constitute an azoonotic transmission cycle. DTU III has also been isolated from humans^17^ ^,^ ^23^ ^,^ ^24^ ^,^ ^25^ ^,^ ^27^ ^,^ ^32^ ^,^ ^35^ ^-^ ^37^. This genotype shows broad distribution among vectors and both wild and anthropogenic mammalian hosts. Its actual prevalence in northeastern Brazil may be underestimated, as DTU III is believed to circulate predominantly in sylvatic cycles in Brazil and neighboring countries. Still, it is rarely reported in human cases. This DTU is almost exclusively associated with transmission cycles involving fossorial mammals, including marsupials^25^ ^,^ ^27^ ^,^ ^38^.

DTU III was reported for the first time in humans in the state of RN, where it was associated with the indeterminate clinical form of Chagas disease^25^. It has also been identified in the state of Minas Gerais^34^. The detection of this DTU in both *Triatoma brasiliensis* and *P. lutzi* suggests a potential for wider distribution of this genotype in the region, particularly in the absence of continuous entomological surveillance and effective vector control. These vector species may serve as epidemiological links between sylvatic and domestic transmission cycles in specific geographic areas, facilitating the spread of this genotype across different environments^17^ ^,^ ^27^. DTU III has been identified in various contexts, including the western Brazilian Amazon, where it has been linked to contamination of *açaí* juice^39^; in domestic mammals in Argentina and Paraguay; and in wild mammals, domestic dogs, and humans in Brazil^12^ ^,^ ^38^. Some studies suggest that DTU III may have originated from an ancestral hybridization between DTUs I and II, resulting in low genetic diversity and a likely preference for specific mammalian hosts^40^ ^,^ ^41^. The high frequency of DTU III in *T. brasiliensis* in RN supports its *dual* epidemiological role in both sylvatic and potential domestic transmission cycles^26^ ^,^ ^27^. Although the triatomine species associated with this DTU are not yet fully characterized, infections have been reported in both *P. lutzi* and *T. brasiliensis* ^25^ ^,^ ^27^.

The co-occurrence of DTUs I and II in the same geographic areas indicates that DTU III is capable of coexisting with other genotypes in environments where sylvatic and peridomestic transmission cycles overlap-epidemiological patterns observed in the region studied^17^ ^,^ ^23^ ^-^ ^27^. This overlap may be facilitated by the ecological characteristics of the northeastern Brazilian biome. In this region, *P. lutzi* infected with DTU III participates in the sylvatic cycle. In contrast, *T. brasiliensis*, infected with DTUs II and III, can contribute to both sylvatic and peridomestic transmission. The presence of mammalian reservoirs and triatomine vectors near human settlements removes barriers to the introduction of DTU III into anthropogenic environments^25^ ^,^ ^26^. In our study area, the Caatinga biome is undergoing desertification, a trend also observed in the neighboring state of Ceará^42^, possibly linked to climate change^43^. This desertification is driven by both natural and anthropogenic factors^18^ including agricultural expansion, livestock farming, and surface mining. These activities have accelerated environmental degradation, leading to soil salinization, infertility, erosion, and significant biodiversity or migration of local fauna to neighboring stable environments, favoring the proximity of sylvatic triatomines and human habitations^18^.

In this context, the sympatric presence of two or more DTUs within a single mammalian host and/or triatomine vector can lead to the formation of multiclonal strains^44^ and, consequently, the emergence of new favorable recombinant genotypes^45^. Mixed infections involving DTUs I+II have been identified in *T. brasiliensis* in both sylvatic and peridomestic environments^17^ ^,^ ^23^. The occurrence of co-infections with *T. cruzi* and *T. rangeli* genotypes further underscores the importance of strengthening differential diagnostic efforts, as *T. rangeli*, although nonpathogenic, shares a similar geographic distribution and is transmitted by the same vectors as *T. cruzi*. Cross-reactivity may also occur in serological tests for chronic infection, complicating diagnosis^23^ ^,^ ^46^.

The intradomestic transmission cycle of *T. cruzi* arises from interactions between humans and vectors, often driven by social and ecological changes caused by human activity. These changes facilitate the colonization of artificial ecotopes by triatomines^18^. *T. brasiliensis* plays a central role in this epidemiological context, as it is the most frequently infected species in the studied area. It has been found with monoinfections of DTU I^17^ ^,^ ^22^ ^-^ ^24^, DTU II^17^ ^,^ ^22^ ^,^ ^23^ ^,^ ^27^ ^,^ ^32^ or DTU III^17^ ^,^ ^23^ ^,^ ^24^ ^,^ ^27^ ^,^ ^32^. Mixed infections of DTUs I+II^17^ ^,^ ^23^ and DTUs II+II+TrA or DTU/species I+TrA have also been reported^23^. *T. brasiliensis* and *T. pseudomaculata* are native triatomine species, with their dispersal epicenter located in northeastern Brazil^18^. In its natural habitat, *T. brasiliensis* is commonly found among rocky outcrops and cacti, feeding primarily on rodents. These rodents, such as *Pilosocereus gounellei*, often share the same ecotopes with the vector species^47^ ^-^ ^49^.

In artificial ecotopes, *T. brasiliensis* can infest and establish colonies near human dwellings, feeding on a variety of domestic and peridomestic animals and acting as a bridge between sylvatic and anthropogenic environments^42^ ^,^ ^50^ ^-^ ^52^. *T. brasiliensis* is the most studied member of the *brasiliensis* complex due to its strong capacity to invade and/or colonize households. This behavior enables transmission through the classic vectorial route, as well as via ingestion of food contaminated with infected feces^17^ ^,^ ^29^ ^,^ ^32^ ^,^ ^47^ ^-^ ^48^. The behavior of this vector, combined with its high infection rates and insufficient vector control measures, facilitates the occurrence of oral transmission outbreaks involving *T. brasiliensis*, such as the 2016 outbreak in the municipality of Marcelino Vieira in RN^29^. This remains an ongoing public health issue in the state, where there continues to be a risk of recolonization in both peridomestic and intradomestic environments^53^.

Previous studies^50^, reported triatomine populations with extremely high *T. cruzi* prevalence (>94%) that fed exclusively on cavies-one of which (MV188P) was collected near a sugarcane mill linked to a recent Chagas disease outbreak. Furthermore, another study^54^, showed that individuals feeding on *Didelphis* spp. and *G. spixii* carried higher parasitic loads, reinforcing their roles as important reservoirs. In Caicó, sylvatic *T. cruzi* -infected insects were also found to feed almost exclusively on *Kerodon rupestris*, another caviid species, further suggesting the importance of this group of rodents in maintaining sylvatic transmission cycles^55^.

The species *P. lutzi*, *T. pseudomaculata*, and *T. petrocchiae* are additional *T. cruzi* vectors identified in the state of RN, and they may play important roles in sustaining parasite transmission cycles^17^ ^,^ ^56^ *. P. lutzi is* restricted to the Caatinga biome in northeastern Brazil, where it inhabits both peridomestic and domestic environments. It is considered an important species for maintaining the enzootic transmission cycle of *T. cruzi*, as it feeds on a wide range of animal hosts^57^. Nymphs of this species have been detected in both peridomestic and intradomestic environments, indicating its capacity to colonize anthropogenic habitats, where it shows high rates of natural infection with *T. cruzi* ^17^ ^,^ ^26^ ^,^ ^27^ ^,^ ^32^. These findings suggest that several triatomine species may currently be undergoing a process of domiciliation in the region, potentially driven by climate change and biodiversity loss, resulting in increased dispersion as they seek new blood meal sources. This behavior leads to greater interaction with domestic animals and humans^17^.

Various mammalian species infected with *T. cruzi* in RN, such as *E. sexcinctus*, *G. spixii*, and Ovis *aries*, are also essential for the establishment and maintenance of the parasite in anthropogenic environments, supporting the potential for active vector-borne transmission^12^ ^,^ ^25^ ^,^ ^30^ ^,^ ^35^ ^,^ ^37^. However, the most critical factor in sustaining transmission in endemic areas appears to be the ecological plasticity of vector species. Their ability to colonize both sylvatic and anthropogenic ecotopes allows them, and the synanthropic mammals they feed on-to inhabit the same spaces as humans. This expansion and adaptability of triatomine vectors represent one of the greatest challenges for entomological surveillance, which must be ongoing and continuous.

## CONCLUSION

This study demonstrates that the detection of *Trypanosoma cruzi* genotypes across a range of mammalian hosts, including humans, and multiple vector species underscores the persistent risk of active transmission to human populations. These findings reinforce the urgent need for sustained and continuous entomological surveillance in the state of Rio Grande do Norte, Brazil. Future research will be essential to deepen our understanding of infection dynamics and to enable comparative analyses based on the baseline data established here. Moreover, in regions where the epidemiological patterns of Chagas disease are unstable, primarily due to anthropogenic activities and the exploitation of natural resources -such insights are critical for informing the development and implementation of effective strategies for the control and prevention of *T. cruzi* infection.
