# Supplementary material for: Distribution, host origin, and transmission cycles of Trypanosoma cruzi genotypes in the State of Rio Grande do Norte, Brazil
Source: Rev Soc Bras Med Trop. 2025 Aug 8;58:e0063-2025. doi: 10.1590/0037-8682-0063-2025 (PMC12333620; doi:10.1590/0037-8682-0063-2025)
Supplement: Supplementary file 1 [file 1678-9849-rsbmt-58-e0063-2025-supp1.pdf]

Supplementary: The principal characteristics of the studies included in the systematic revision.

dbID#	Article ID	Author/Year	Number of isolates	Intervention group	Isolate name	DTU
db160	11	Martins et al., 2015	20		Es18	DTUIII
db161	11	Martins et al., 2015	20		Gs3	DTUIII
db181	11	Martins et al., 2015	20		105	DTUIII
db253	11	Martins et al., 2015	20		CBS195	DTUIII
db254	11	Martins et al., 2015	20		SM76	DTUIII
db255	11	Martins et al., 2015	20		PI0213	DTUIII
db256	11	Martins et al., 2015	20		PI0812	DTUIII
db185	3	Lima-Neiva et al., 2021	28	Pharmacology		DTUI
db248	3	Lima-Neiva et al., 2021	28	Pharmacology		DTUII
db249	3	Lima-Neiva et al., 2021	28	Pharmacology		DTUI
db286	3	Lima-Neiva et al., 2021	28	Pharmacology		DTUI
db287	3	Lima-Neiva et al., 2021	28	Pharmacology		DTUII
db288	3	Lima-Neiva et al., 2021	28	Pharmacology		DTUII
db246	1	Araújo-Neto et al., 2023	1	Analysis of risk	SH022	DTUII
db257	7	Pereira et al., 2020	1		RN 25	DTUII
db258	13	Camara et al., 2010	25		RN01	DTUII
db259	13	Camara et al., 2010	25		RN03	DTUII
db260	13	Camara et al., 2010	25		RN06	DTUII
db261	13	Camara et al., 2010	25		RN02	DTUIII
db262	13	Camara et al., 2010	25		RN04	DTUIII
db263	13	Camara et al., 2010	25		RN05	DTUIII
db264	13	Camara et al., 2010	25		RN24–481	DTUII
db267	5	Honorato et al., 2021	33		MV3Tb	DTUI
db268	5	Honorato et al., 2021	33		MV5Tb	DTUI
db269	6	Queiroga et al., 2021	1		PL1.10.16	DTUIII
db272	13	Camara et al., 2010	25		RN26–238	DTUII
db273	5	Honorato et al., 2021	33		MV1Tb	DTUI
db002	12	Camara et al., 2013	24		02TbM/SNN	DTUII

db010	12	Camara et al., 2013	24		12PIM/SNN	DTUIII
db011	12	Camara et al., 2013	24		04TbM/SNN	DTUIII
db012	12	Camara et al., 2013	24		05TbM/SNN	DTUIII
db153	12	Camara et al., 2013	24		18PIM/CBS	DTUIII
db154	12	Camara et al., 2013	24		19PIM/CBS	DTUIII
db155	12	Camara et al., 2013	24		07PIM/SNN	DTUIII
db156	12	Camara et al., 2013	24		09PIM/SNN	DTUIII
db157	12	Camara et al., 2013	24		10PIM/SNN	DTUIII
db158	12	Camara et al., 2013	24		11PIM/SNN	DTUIII
db184	12	Camara et al., 2013	24		17HP/CBS	DTUII
db265	12	Camara et al., 2013	24		15HM/CBS	DTUII
db266	12	Camara et al., 2013	24		16HP/CBS	DTUII
db270	12	Camara et al., 2013	24		13HP/CBS	DTUII
db271	12	Camara et al., 2013	24		14HM/CBS	DTUII
db278	12	Camara et al., 2013	24		20HM/CBS	DTUII
db279	12	Camara et al., 2013	24		21HP/CBS	DTUII
db280	12	Camara et al., 2013	24		08HP/CBS	DTUII
db281	12	Camara et al., 2013	24		01TbM/CAC	DTUII
db282	12	Camara et al., 2013	24		03TbM/SNN	DTUII
db283	12	Camara et al., 2013	24		06TbP/SNN	DTUII
db013	7	Pereira et al., 2020	1		RN 25	DTUII
db014	13	Camara et al., 2010	25		RN15–722	DTUII
db015	13	Camara et al., 2010	25		RN16–804	DTUII
db017	3	Lima-Neiva et al., 2021	28	Pharmacology		DTUI
db018	3	Lima-Neiva et al., 2021	28	Pharmacology		DTUI
db019	3	Lima-Neiva et al., 2021	28	Pharmacology		DTUII
db180	13	Camara et al., 2010	25		RN17–792	DTUII
db230	6	Queiroga et al., 2021	1		PL1.10.15	DTUIII
db234	13	Camara et al., 2010	25		RN20–745	DTUII
db235	13	Camara et al., 2010	25		RN21–782	DTUII
db236	13	Camara et al., 2010	25		RN23–462	DTUII
db237	5	Honorato et al., 2021	33		CA168Tb	DTUI
db238	5	Honorato et al., 2021	33		CA171Tb	DTUI
db239	5	Honorato et al., 2021	33		CBS1Tb	DTUI
db240	5	Honorato et al., 2021	33		CBS4Tb	DTUI

db241	5	Honorato et al., 2021	33		ODB3Tb	DTUI
db242	5	Honorato et al., 2021	33		SC1Tb	DTUI
db243	1	Araújo-Neto et al., 2023	1	Analysis of risk	SH022	DTUII
db289	3	Lima-Neiva et al., 2021	28	Pharmacology		DTUI
db290	3	Lima-Neiva et al., 2021	28	Pharmacology		DTUI
db291	3	Lima-Neiva et al., 2021	28	Pharmacology		DTUI
db231	11	Martins et al., 2015	20		3188	DTUI
db232	11	Martins et al., 2015	20		CBS 202	DTUI
db233	11	Martins et al., 2015	20		240	DTUII
db183	11	Martins et al., 2015	20		1317	DTUII
db274	11	Martins et al., 2015	20		2934	DTUII
db275	11	Martins et al., 2015	20		3973a	DTUII
db276	11	Martins et al., 2015	20		RN26	DTUII
db277	11	Martins et al., 2015	20		RN79	DTUII
db001	11	Martins et al., 2015	20		SM73	DTUII
db006	11	Martins et al., 2015	20		1816	DTUI
db008	11	Martins et al., 2015	20		2137	DTUI
db009	11	Martins et al., 2015	20		2549	DTUI
db179	11	Martins et al., 2015	20		1150	DTUI
db007	13	Camara et al., 2010	25		RN14–838	DTUII
db016	3	Lima-Neiva et al., 2021	28	Pharmacology		DTUI
db020	3	Lima-Neiva et al., 2021	28	Pharmacology		DTUI
db021	3	Lima-Neiva et al., 2021	28	Pharmacology		DTUII
db149	7	Pereira et al., 2020	1		RN 25	DTUII
db150	13	Camara et al., 2010	25		RN22–985	DTUI
db151	13	Camara et al., 2010	25		RN08	DTUII
db152	13	Camara et al., 2010	25		RN13–611	DTUII
db220	5	Honorato et al., 2021	33		CA95Tb	DTUI
db221	5	Honorato et al., 2021	33		CA105Tb	DTUI
db222	5	Honorato et al., 2021	33		CA167Tb	DTUI
db226	6	Queiroga et al., 2021	1		PL1.10.14	DTUIII
db227	5	Honorato et al., 2021	33		CA93Tb	DTUI
db244	1	Araújo-Neto et al., 2023	1	Analysis of risk	SH022	DTUII
db284	3	Lima-Neiva et al., 2021	28	Pharmacology		DTUI

db193	10	Barbosa-Silva et al., 2016	16		RN02	DTUIII
db194	10	Barbosa-Silva et al., 2016	16		RN03	DTUII
db195	10	Barbosa-Silva et al., 2016	16		RN04	DTUIII
db196	10	Barbosa-Silva et al., 2016	16		RN05	DTUIII
db197	10	Barbosa-Silva et al., 2016	16		RN06	DTUII
db214	10	Barbosa-Silva et al., 2016	16		RN10	DTUIII
db215	10	Barbosa-Silva et al., 2016	16		RN11	DTUIII
db216	10	Barbosa-Silva et al., 2016	16		RN12	DTUIII
db217	10	Barbosa-Silva et al., 2016	16		RN213	DTUIII
db218	10	Barbosa-Silva et al., 2016	16		RN812	DTUIII
db219	10	Barbosa-Silva et al., 2016	16		RN01	DTUII
db223	10	Barbosa-Silva et al., 2016	16		RN18	DTUIII
db224	10	Barbosa-Silva et al., 2016	16		RN19	DTUIII
db225	10	Barbosa-Silva et al., 2016	16		RN07	DTUIII
db228	10	Barbosa-Silva et al., 2016	16		RN08	DTUIII
db229	10	Barbosa-Silva et al., 2016	16		RN09	DTUIII
db072	8	Lima-Oliveira et al., 2020	28			DTUI
db187	8	Lima-Oliveira et al., 2020	28			DTUI
db188	8	Lima-Oliveira et al., 2020	28			DTUI
db189	8	Lima-Oliveira et al., 2020	28			DTUI
db190	8	Lima-Oliveira et al., 2020	28			DTUIII
db191	8	Lima-Oliveira et al., 2020	28			DTUIII
db192	8	Lima-Oliveira et al., 2020	28			DTUIII
db198	8	Lima-Oliveira et al., 2020	28			DTUI
db199	8	Lima-Oliveira et al., 2020	28			DTUI
db200	8	Lima-Oliveira et al., 2020	28			DTUI
db201	8	Lima-Oliveira et al., 2020	28			DTUI
db202	8	Lima-Oliveira et al., 2020	28			DTUI
db203	8	Lima-Oliveira et al., 2020	28			DTUI
db204	8	Lima-Oliveira et al., 2020	28			DTUI
db205	8	Lima-Oliveira et al., 2020	28			DTUI
db206	8	Lima-Oliveira et al., 2020	28			DTUI
db207	8	Lima-Oliveira et al., 2020	28			DTUI
db208	8	Lima-Oliveira et al., 2020	28			DTUI

db209	8	Lima-Oliveira et al., 2020	28			DTUI
db210	8	Lima-Oliveira et al., 2020	28			DTUI
db211	8	Lima-Oliveira et al., 2020	28			DTUI
db212	8	Lima-Oliveira et al., 2020	28			DTUI
db213	8	Lima-Oliveira et al., 2020	28			DTUI
db250	8	Lima-Oliveira et al., 2020	28			DTUI
db251	8	Lima-Oliveira et al., 2020	28			DTUI
db252	8	Lima-Oliveira et al., 2020	28			DTUI
db073	12	Camara et al., 2013	24		28H/ANG	DTUI
db074	12	Camara et al., 2013	24		29H/CAC	DTUI
db075	12	Camara et al., 2013	24		22HM/CBS	DTUI
db247	9	Ribeiro et al., 2018	1		TCC863	DTUIII
db004	4	Valença-Barbosa et al., 2021	TcI+TcII+TrA (n:1)			DTUIII
db038	4	Valença-Barbosa et al., 2021	TcI+TcII+TrA (n:1)			DTUI + II
db039	4	Valença-Barbosa et al., 2021	TcI+TcII+TrA (n:1)			DTUI + Tcr
db040	4	Valença-Barbosa et al., 2021	TcI+TcII+TrA (n:1)			DTUI + Tcr
db041	4	Valença-Barbosa et al., 2021	TcI+TcII+TrA (n:1)			DTUI + II + Tcr
db042	4	Valença-Barbosa et al., 2021	TcII (n:36)			DTUI
db043	4	Valença-Barbosa et al., 2021	TcIII (n:2)			DTUI
db044	4	Valença-Barbosa et al., 2021	TcI+TcII (n:5)			DTUI
db045	4	Valença-Barbosa et al., 2021	TcI+TrA (n:2)			DTUI
db046	4	Valença-Barbosa et al., 2021	TcI+TcII+TrA (n:1)			DTUI
db047	4	Valença-Barbosa et al., 2021	TcI+TcII+TrA (n:1)			DTUI
db048	4	Valença-Barbosa et al., 2021	TcI+TcII+TrA (n:1)			DTUI
db049	4	Valença-Barbosa et al., 2021	TcI+TcII+TrA (n:1)			DTUI
db050	4	Valença-Barbosa et al., 2021	TcI+TcII+TrA (n:1)			DTUI
db051	4	Valença-Barbosa et al., 2021	TcI+TcII+TrA (n:1)			DTUI
db052	4	Valença-Barbosa et al., 2021	TcI+TcII+TrA (n:1)			DTUI
db053	4	Valença-Barbosa et al., 2021	TcI+TcII+TrA (n:1)			DTUI
db054	4	Valença-Barbosa et al., 2021	TcI+TcII+TrA (n:1)			DTUI
db055	4	Valença-Barbosa et al., 2021	TcI+TcII+TrA (n:1)			DTUI
db056	4	Valença-Barbosa et al., 2021	TcI+TcII+TrA (n:1)			DTUI
db057	4	Valença-Barbosa et al., 2021	TcI+TcII+TrA (n:1)			DTUI
db058	4	Valença-Barbosa et al., 2021	TcI+TcII+TrA (n:1)			DTUI

db059	4	Valença-Barbosa et al., 2021	TcI+TcII+TrA (n:1)			DTUI
db060	4	Valença-Barbosa et al., 2021	TcI+TcII+TrA (n:1)			DTUI
db061	4	Valença-Barbosa et al., 2021	TcI+TcII+TrA (n:1)			DTUI
db062	4	Valença-Barbosa et al., 2021	TcI+TcII+TrA (n:1)			DTUI
db063	4	Valença-Barbosa et al., 2021	TcI+TcII+TrA (n:1)			DTUI
db064	4	Valença-Barbosa et al., 2021	TcI+TcII+TrA (n:1)			DTUI
db065	4	Valença-Barbosa et al., 2021	TcI+TcII+TrA (n:1)			DTUI
db066	4	Valença-Barbosa et al., 2021	TcI+TcII+TrA (n:1)			DTUI
db067	4	Valença-Barbosa et al., 2021	TcI+TcII+TrA (n:1)			DTUI
db068	4	Valença-Barbosa et al., 2021	TcI+TcII+TrA (n:1)			DTUI
db069	4	Valença-Barbosa et al., 2021	TcI+TcII+TrA (n:1)			DTUI
db070	4	Valença-Barbosa et al., 2021	TcI+TcII+TrA (n:1)			DTUI
db071	4	Valença-Barbosa et al., 2021	TcI+TcII+TrA (n:1)			DTUI
db076	4	Valença-Barbosa et al., 2021	TcI+TcII+TrA (n:1)			DTUI
db077	4	Valença-Barbosa et al., 2021	TcI+TcII+TrA (n:1)			DTUI
db078	4	Valença-Barbosa et al., 2021	TcI+TcII+TrA (n:1)			DTUI
db079	4	Valença-Barbosa et al., 2021	TcI+TcII+TrA (n:1)			DTUI
db080	4	Valença-Barbosa et al., 2021	TcI+TcII+TrA (n:1)			DTUI
db081	4	Valença-Barbosa et al., 2021	TcI+TcII+TrA (n:1)			DTUI
db082	4	Valença-Barbosa et al., 2021	TcI+TcII+TrA (n:1)			DTUI
db083	4	Valença-Barbosa et al., 2021	TcI+TcII+TrA (n:1)			DTUI
db084	4	Valença-Barbosa et al., 2021	TcI+TcII+TrA (n:1)			DTUI
db085	4	Valença-Barbosa et al., 2021	TcI+TcII+TrA (n:1)			DTUI
db086	4	Valença-Barbosa et al., 2021	TcI+TcII+TrA (n:1)			DTUI
db087	4	Valença-Barbosa et al., 2021	TcI+TcII+TrA (n:1)			DTUI
db088	4	Valença-Barbosa et al., 2021	TcI+TcII+TrA (n:1)			DTUI
db089	4	Valença-Barbosa et al., 2021	TcI+TcII+TrA (n:1)			DTUI
db090	4	Valença-Barbosa et al., 2021	TcI+TcII+TrA (n:1)			DTUI
db091	4	Valença-Barbosa et al., 2021	TcI+TcII+TrA (n:1)			DTUI
db092	4	Valença-Barbosa et al., 2021	TcI+TcII+TrA (n:1)			DTUI
db093	4	Valença-Barbosa et al., 2021	TcI+TcII+TrA (n:1)			DTUI
db094	4	Valença-Barbosa et al., 2021	TcI+TcII+TrA (n:1)			DTUI
db095	4	Valença-Barbosa et al., 2021	TcI+TcII+TrA (n:1)			DTUI
db096	4	Valença-Barbosa et al., 2021	TcI+TcII+TrA (n:1)			DTUI

db097	4	Valença-Barbosa et al., 2021	TcI+TcII+TrA (n:1)			DTUI
db098	4	Valença-Barbosa et al., 2021	TcI+TcII+TrA (n:1)			DTUI
db099	4	Valença-Barbosa et al., 2021	TcI+TcII+TrA (n:1)			DTUI
db100	4	Valença-Barbosa et al., 2021	TcI+TcII+TrA (n:1)			DTUI
db101	4	Valença-Barbosa et al., 2021	TcI+TcII+TrA (n:1)			DTUI
db102	4	Valença-Barbosa et al., 2021	TcI+TcII+TrA (n:1)			DTUI
db103	4	Valença-Barbosa et al., 2021	TcI+TcII+TrA (n:1)			DTUI
db104	4	Valença-Barbosa et al., 2021	TcI+TcII+TrA (n:1)			DTUI
db105	4	Valença-Barbosa et al., 2021	TcI+TcII+TrA (n:1)			DTUI
db106	4	Valença-Barbosa et al., 2021	TcI+TcII+TrA (n:1)			DTUI
db107	4	Valença-Barbosa et al., 2021	TcI+TcII+TrA (n:1)			DTUI
db108	4	Valença-Barbosa et al., 2021	TcI+TcII+TrA (n:1)			DTUII
db109	4	Valença-Barbosa et al., 2021	TcI+TcII+TrA (n:1)			DTUII
db110	4	Valença-Barbosa et al., 2021	TcI+TcII+TrA (n:1)			DTUII
db111	4	Valença-Barbosa et al., 2021	TcI+TcII+TrA (n:1)			DTUII
db112	4	Valença-Barbosa et al., 2021	TcI+TcII+TrA (n:1)			DTUII
db113	4	Valença-Barbosa et al., 2021	TcI+TcII+TrA (n:1)			DTUII
db114	4	Valença-Barbosa et al., 2021	TcI+TcII+TrA (n:1)			DTUII
db115	4	Valença-Barbosa et al., 2021	TcI+TcII+TrA (n:1)			DTUII
db116	4	Valença-Barbosa et al., 2021	TcI+TcII+TrA (n:1)			DTUII
db117	4	Valença-Barbosa et al., 2021	TcI+TcII+TrA (n:1)			DTUII
db118	4	Valença-Barbosa et al., 2021	TcI+TcII+TrA (n:1)			DTUII
db119	4	Valença-Barbosa et al., 2021	TcI+TcII+TrA (n:1)			DTUII
db120	4	Valença-Barbosa et al., 2021	TcI+TcII+TrA (n:1)			DTUII
db121	4	Valença-Barbosa et al., 2021	TcI+TcII+TrA (n:1)			DTUII
db122	4	Valença-Barbosa et al., 2021	TcI+TcII+TrA (n:1)			DTUII
db123	4	Valença-Barbosa et al., 2021	TcI+TcII+TrA (n:1)			DTUII
db124	4	Valença-Barbosa et al., 2021	TcI+TcII+TrA (n:1)			DTUII
db125	4	Valença-Barbosa et al., 2021	TcI+TcII+TrA (n:1)			DTUII
db126	4	Valença-Barbosa et al., 2021	TcI+TcII+TrA (n:1)			DTUII
db127	4	Valença-Barbosa et al., 2021	TcI+TcII+TrA (n:1)			DTUII
db128	4	Valença-Barbosa et al., 2021	TcI+TcII+TrA (n:1)			DTUI
db129	4	Valença-Barbosa et al., 2021	TcI+TcII+TrA (n:1)			DTUI
db130	4	Valença-Barbosa et al., 2021	TcI+TcII+TrA (n:1)			DTUII

db131	4	Valença-Barbosa et al., 2021	TcI+TcII+TrA (n:1)			DTUII
db132	4	Valença-Barbosa et al., 2021	TcI+TcII+TrA (n:1)			DTUII
db133	4	Valença-Barbosa et al., 2021	TcI+TcII+TrA (n:1)			DTUII
db134	4	Valença-Barbosa et al., 2021	TcI+TcII+TrA (n:1)			DTUII
db135	4	Valença-Barbosa et al., 2021	TcI+TcII+TrA (n:1)			DTUII
db136	4	Valença-Barbosa et al., 2021	TcI+TcII+TrA (n:1)			DTUII
db137	4	Valença-Barbosa et al., 2021	TcI+TcII+TrA (n:1)			DTUII
db138	4	Valença-Barbosa et al., 2021	TcI+TcII+TrA (n:1)			DTUII
db139	4	Valença-Barbosa et al., 2021	TcI+TcII+TrA (n:1)			DTUI
db140	4	Valença-Barbosa et al., 2021	TcI+TcII+TrA (n:1)			DTUII
db141	4	Valença-Barbosa et al., 2021	TcI+TcII+TrA (n:1)			DTUII
db142	4	Valença-Barbosa et al., 2021	TcI+TcII+TrA (n:1)			DTUII
db143	4	Valença-Barbosa et al., 2021	TcI+TcII+TrA (n:1)			DTUII
db144	4	Valença-Barbosa et al., 2021	TcI+TcII+TrA (n:1)			DTUII
db145	4	Valença-Barbosa et al., 2021	TcI+TcII+TrA (n:1)			DTUI + II
db146	4	Valença-Barbosa et al., 2021	TcI+TcII+TrA (n:1)			DTUI + II
db147	4	Valença-Barbosa et al., 2021	TcI+TcII+TrA (n:1)			DTUI + II
db148	4	Valença-Barbosa et al., 2021	TcI+TcII+TrA (n:1)			DTUI + II
db162	4	Valença-Barbosa et al., 2021	TcI+TcII+TrA (n:1)			DTUIII
db164	4	Valença-Barbosa et al., 2021	TcI+TcII+TrA (n:1)			DTU II
db165	4	Valença-Barbosa et al., 2021	TcI+TcII+TrA (n:1)			DTUII
db003	3	Lima-Neiva et al., 2021	28	Pharmacology		DTUI
db022	6	Queiroga et al., 2021	1		PL1.10.17	DTUIII
db023	5	Honorato et al., 2021	33		CA103Tb	DTUI + II
db024	5	Honorato et al., 2021	33		CA109Tb	DTUI + II
db025	3	Lima-Neiva et al., 2021	28			DTUI
db026	3	Lima-Neiva et al., 2021	28			DTUI
db027	3	Lima-Neiva et al., 2021	28			DTUI
db028	3	Lima-Neiva et al., 2021	28			DTUI
db029	3	Lima-Neiva et al., 2021	28			DTUII
db030	3	Lima-Neiva et al., 2021	28			DTUII
db031	3	Lima-Neiva et al., 2021	28			DTUI
db032	3	Lima-Neiva et al., 2021	28			DTUII
db033	3	Lima-Neiva et al., 2021	28			DTUII

db034	13	Camara et al., 2010	25		RN19	DTUIII
db163	5	Honorato et al., 2021	33		CBS55Tb	DTUIII
db166	5	Honorato et al., 2021	33		ODB1Tp	DTUI
db167	5	Honorato et al., 2021	33		SNN1PI	DTUIII
db168	5	Honorato et al., 2021	33		CBS56Tb	DTUIII
db169	5	Honorato et al., 2021	33		MV5Tb	DTUII
db170	5	Honorato et al., 2021	33		JC12Tb	DTUIII
db171	5	Honorato et al., 2021	33		JC13Tb	DTUIII
db172	5	Honorato et al., 2021	33		JC14Tb	DTUIII
db173	5	Honorato et al., 2021	33		JC15Tb	DTUIII
db174	5	Honorato et al., 2021	33		MV5Tb	DTUII
db175	5	Honorato et al., 2021	33		JC16Tb	DTUIII
db176	5	Honorato et al., 2021	33		JC17Tb	DTUIII
db177	5	Honorato et al., 2021	33		CA92Tb	DTUI + II
db178	5	Honorato et al., 2021	33		CA97Tb	DTUI + II
db182	5	Honorato et al., 2021	33		SNN4Tb	DTUII
db186	3	Lima-Neiva et al., 2021	28			DTUI
db245	1	Araújo-Neto et al., 2023	1	Analysis of risk	SH022	DTUII
db285	3	Lima-Neiva et al., 2021	28	Pharmacology		DTUI
db292	7	Pereira et al., 2020	1		RN 25	DTUII
db293	13	Camara et al., 2010	25		RN07	DTUIII
db294	13	Camara et al., 2010	25		RN09	DTUIII
db295	13	Camara et al., 2010	25		RN10	DTUIII
db296	13	Camara et al., 2010	25		RN11	DTUIII
db297	13	Camara et al., 2010	25		RN12	DTUIII
db298	13	Camara et al., 2010	25		RN18	DTUIII
db005	4	Valença-Barbosa et al., 2021	TcI (n:70)			DTUI
db035	4	Valença-Barbosa et al., 2021	116			DTUI
db036	4	Valença-Barbosa et al., 2021	116			DTUI
db037	4	Valença-Barbosa et al., 2021	116			DTUI
db159	2	Hickson et el., 2022	1	Pharmacology	PEBA18	DTUIII

Source: prepared by the authors.
Legend: X is equivalent to information not available in the article.

Genetic characterization protocol
(Souto&Zingales, 1993, Souto et al, 1996) SL-IR- Primers: TcIII & UTCC (Burgos al al., 2007)
(Souto&Zingales, 1993, Souto et al, 1996) SL-IR- Primers: TcIII & UTCC (Burgos al al., 2007)
(Souto&Zingales, 1993, Souto et al, 1996) SL-IR- Primers: TcIII & UTCC (Burgos al al., 2007)
(Souto&Zingales, 1993, Souto et al, 1996) SL-IR- Primers: TcIII & UTCC (Burgos al al., 2007)
(Souto&Zingales, 1993, Souto et al, 1996) SL-IR- Primers: TcIII & UTCC (Burgos al al., 2007)
(Souto&Zingales, 1993, Souto et al, 1996) SL-IR- Primers: TcIII & UTCC (Burgos al al., 2007)
(Souto&Zingales, 1993, Souto et al, 1996) SL-IR- Primers: TcIII & UTCC (Burgos al al., 2007)
24Sα rDNA- Primers D71 & D72 (Souto&Zingales, 1993)
24Sα rDNA- Primers D71 & D72 (Souto&Zingales, 1993)
24Sα rDNA- Primers D71 & D72 (Souto&Zingales, 1993)
24Sα rDNA- Primers D71 & D72 (Souto&Zingales, 1993)
24Sα rDNA- Primers D71 & D72 (Souto&Zingales, 1993)
24Sα rDNA- Primers D71 & D72 (Souto&Zingales, 1993)
24Sα rDNA- Primers D71 & D72 (Souto&Zingales, 1993, Souto et al, 1996)
24Sα rDNA- Primers D71 & D72 (Souto&Zingales, 1993, Souto et al, 1996)
24Sα rDNA- Primers D71 & D72 (Souto&Zingales, 1993, Souto et al, 1996)
24Sα rDNA- Primers D71 & D72 (Souto&Zingales, 1993, Souto et al, 1996)
24Sα rDNA- Primers D71 & D72 (Souto&Zingales, 1993, Souto et al, 1996)
24Sα rDNA- Primers D71 & D72 (Souto&Zingales, 1993, Souto et al, 1996)
24Sα rDNA- Primers D71 & D72 (Souto&Zingales, 1993, Souto et al, 1996)
24Sα rDNA- Primers D71 & D72 (Souto&Zingales, 1993, Souto et al, 1996)
24Sα rDNA- Primers D71 & D72 (Souto&Zingales, 1993, Souto et al, 1996)
24Sα rDNA- Primers D71 & D72 (Souto&Zingales, 1993, Souto et al, 1996)
24Sα rDNA- Primers D71 & D72 (Souto&Zingales, 1993, Souto et al, 1996)
24Sα rDNA- Primers D71 & D72 (Souto&Zingales, 1993, Souto et al, 1996)
24Sα rDNA- Primers D71 & D72 (Souto&Zingales, 1993, Souto et al, 1996)
24Sα rDNA- Primers D71 & D72 (Souto&Zingales, 1993, Souto et al, 1996)
Camara et al., 2010

Camara et al., 2010
Camara et al., 2010
Camara et al., 2010
Camara et al., 2010
Camara et al., 2010
Camara et al., 2010
Camara et al., 2010
Camara et al., 2010
Camara et al., 2010
Camara et al., 2010
Camara et al., 2010
Camara et al., 2010
Camara et al., 2010
Camara et al., 2010
Camara et al., 2010
Camara et al., 2010
Camara et al., 2010
Camara et al., 2010
Camara et al., 2010
Camara et al., 2010
COII – primers: Tcmit-10, Tcmit-21 & AluI (Freitas at al., 2006)
COII – primers: Tcmit-10, Tcmit-21 & AluI (Freitas at al., 2006)
COII – primers: Tcmit-10, Tcmit-21 & AluI (Freitas at al., 2006)
COII – primers: Tcmit-10, Tcmit-21 & AluI (Freitas at al., 2006)
COII – primers: Tcmit-10, Tcmit-21 & AluI (Freitas at al., 2006)
COII – primers: Tcmit-10, Tcmit-21 & AluI (Freitas at al., 2006)
COII – primers: Tcmit-10, Tcmit-21 & AluI (Freitas at al., 2006)
COII – primers: Tcmit-10, Tcmit-21 & AluI (Freitas at al., 2006)
COII – primers: Tcmit-10, Tcmit-21 & AluI (Freitas at al., 2006)
COII – primers: Tcmit-10, Tcmit-21 & AluI (Freitas at al., 2006)
COII – primers: Tcmit-10, Tcmit-21 & AluI (Freitas at al., 2006)
COII – primers: Tcmit-10, Tcmit-21 & AluI (Freitas at al., 2006)
COII – primers: Tcmit-10, Tcmit-21 & AluI (Freitas at al., 2006)
COII – primers: Tcmit-10, Tcmit-21 & AluI (Freitas at al., 2006)
COII – primers: Tcmit-10, Tcmit-21 & AluI (Freitas at al., 2006)

COII – primers: Tcmit-10, Tcmit-21 & AluI (Freitas at al., 2006)
COII – primers: Tcmit-10, Tcmit-21 & AluI (Freitas at al., 2006)
COII – primers: Tcmit-10, Tcmit-21 & AluI (Freitas at al., 2006)
COII – primers: Tcmit-10, Tcmit-21 & AluI (Freitas at al., 2006)
COII – primers: Tcmit-10, Tcmit-21 & AluI (Freitas at al., 2006)
COII – primers: Tcmit-10, Tcmit-21 & AluI (Freitas at al., 2006)
COII – primers: Tcmit-10, Tcmit-21 & AluI (Freitas at al., 2006) 24Sα rDNA- Primers D71 & D72
COII – primers: Tcmit-10, Tcmit-21 & AluI (Freitas at al., 2006) 24Sα rDNA- Primers D71 & D73
COII – primers: Tcmit-10, Tcmit-21 & AluI (Freitas at al., 2006) 24Sα rDNA- Primers D71 & D74
COII – primers: Tcmit-10, Tcmit-21 & AluI (Freitas at al., 2006) 24Sα rDNA- Primers D71 & D75
COII – primers: Tcmit-10, Tcmit-21 & AluI (Freitas at al., 2006) 24Sα rDNA- Primers D71 & D76
COII – primers: Tcmit-10, Tcmit-21 & AluI (Freitas at al., 2006) 24Sα rDNA- Primers D71 & D77
COII – primers: Tcmit-10, Tcmit-21 & AluI (Freitas at al., 2006) 24Sα rDNA- Primers D71 & D78
COII – primers: Tcmit-10, Tcmit-21 & AluI (Freitas at al., 2006) 24Sα rDNA- Primers D71 & D79
COII – primers: Tcmit-10, Tcmit-21 & AluI (Freitas at al., 2006) 24Sα rDNA- Primers D71 & D80
D’Ávila at al., 2009
D’Ávila at al., 2009
D’Ávila at al., 2009
D’Ávila at al., 2009
D’Ávila at al., 2009
D’Ávila at al., 2009
D’Ávila at al., 2009
D’Ávila at al., 2009
D’Ávila at al., 2009
D’Ávila at al., 2009
D’Ávila at al., 2009
D’Ávila at al., 2009
D’Ávila at al., 2009
D’Ávila at al., 2009
D’Ávila at al., 2009
D’Ávila at al., 2009
D’Ávila at al., 2009
D’Ávila at al., 2009
D’Ávila at al., 2009

Degrave et al., 1988
Degrave et al., 1988
Degrave et al., 1988
Degrave et al., 1988
Degrave et al., 1988
Degrave et al., 1988
Degrave et al., 1988
Degrave et al., 1988
Degrave et al., 1988
Degrave et al., 1988
Degrave et al., 1988
Degrave et al., 1988
Degrave et al., 1988
Degrave et al., 1988
Degrave et al., 1988
Degrave et al., 1988
Hamilton et al., 2011 - Primers 121 and 122 (I of Sturm et al. (1989) and Wincker et al. (1994).
Hamilton et al., 2011 - Primers 121 and 122 (I of Sturm et al. (1989) and Wincker et al. (1994).
Hamilton et al., 2011 - Primers 121 and 122 (I of Sturm et al. (1989) and Wincker et al. (1994).
Hamilton et al., 2011 - Primers 121 and 122 (I of Sturm et al. (1989) and Wincker et al. (1994).
Hamilton et al., 2011 - Primers 121 and 122 (I of Sturm et al. (1989) and Wincker et al. (1994).
Hamilton et al., 2011 - Primers 121 and 122 (I of Sturm et al. (1989) and Wincker et al. (1994).
Hamilton et al., 2011 - Primers 121 and 122 (I of Sturm et al. (1989) and Wincker et al. (1994).
Hamilton et al., 2011 - Primers 121 and 122 (I of Sturm et al. (1989) and Wincker et al. (1994).
Hamilton et al., 2011 - Primers 121 and 122 (I of Sturm et al. (1989) and Wincker et al. (1994).
Hamilton et al., 2011 - Primers 121 and 122 (I of Sturm et al. (1989) and Wincker et al. (1994).
Hamilton et al., 2011 - Primers 121 and 122 (I of Sturm et al. (1989) and Wincker et al. (1994).
Hamilton et al., 2011 - Primers 121 and 122 (I of Sturm et al. (1989) and Wincker et al. (1994).
Hamilton et al., 2011 - Primers 121 and 122 (I of Sturm et al. (1989) and Wincker et al. (1994).
Hamilton et al., 2011 - Primers 121 and 122 (I of Sturm et al. (1989) and Wincker et al. (1994).
Hamilton et al., 2011 - Primers 121 and 122 (I of Sturm et al. (1989) and Wincker et al. (1994).
Hamilton et al., 2011 - Primers 121 and 122 (I of Sturm et al. (1989) and Wincker et al. (1994).
Hamilton et al., 2011 - Primers 121 and 122 (I of Sturm et al. (1989) and Wincker et al. (1994).
Hamilton et al., 2011 - Primers 121 and 122 (I of Sturm et al. (1989) and Wincker et al. (1994).

Hamilton et al., 2011 - Primers 121 and 122 (I of Sturm et al. (1989) and Wincker et al. (1994).
Hamilton et al., 2011 - Primers 121 and 122 (I of Sturm et al. (1989) and Wincker et al. (1994).
Hamilton et al., 2011 - Primers 121 and 122 (I of Sturm et al. (1989) and Wincker et al. (1994).
Hamilton et al., 2011 - Primers 121 and 122 (I of Sturm et al. (1989) and Wincker et al. (1994).
Hamilton et al., 2011 - Primers 121 and 122 (I of Sturm et al. (1989) and Wincker et al. (1994).
Hamilton et al., 2011 - Primers 121 and 122 (I of Sturm et al. (1989) and Wincker et al. (1994).
Hamilton et al., 2011 - Primers 121 and 122 (I of Sturm et al. (1989) and Wincker et al. (1994).
Hamilton et al., 2011 - Primers 121 and 122 (I of Sturm et al. (1989) and Wincker et al. (1994).
PCR
PCR
PCR
Primers: m 609F, 706R
Primers:121 (5’ AAATAATGTACGGG(T/G)GAGATGCATGA 3’) & reverse 122 (5’ GGTTCGATTGGGGTTGGTGAATATA 3’)
Primers:121 (5’ AAATAATGTACGGG(T/G)GAGATGCATGA 3’) & reverse 122 (5’ GGTTCGATTGGGGTTGGTGAATATA 3’)
Primers:121 (5’ AAATAATGTACGGG(T/G)GAGATGCATGA 3’) & reverse 122 (5’ GGTTCGATTGGGGTTGGTGAATATA 3’)
Primers:121 (5’ AAATAATGTACGGG(T/G)GAGATGCATGA 3’) & reverse 122 (5’ GGTTCGATTGGGGTTGGTGAATATA 3’)
Primers:121 (5’ AAATAATGTACGGG(T/G)GAGATGCATGA 3’) & reverse 122 (5’ GGTTCGATTGGGGTTGGTGAATATA 3’)
Primers:121 (5’ AAATAATGTACGGG(T/G)GAGATGCATGA 3’) & reverse 122 (5’ GGTTCGATTGGGGTTGGTGAATATA 3’)
Primers:121 (5’ AAATAATGTACGGG(T/G)GAGATGCATGA 3’) & reverse 122 (5’ GGTTCGATTGGGGTTGGTGAATATA 3’)
Primers:121 (5’ AAATAATGTACGGG(T/G)GAGATGCATGA 3’) & reverse 122 (5’ GGTTCGATTGGGGTTGGTGAATATA 3’)
Primers:121 (5’ AAATAATGTACGGG(T/G)GAGATGCATGA 3’) & reverse 122 (5’ GGTTCGATTGGGGTTGGTGAATATA 3’)
Primers:121 (5’ AAATAATGTACGGG(T/G)GAGATGCATGA 3’) & reverse 122 (5’ GGTTCGATTGGGGTTGGTGAATATA 3’)
Primers:121 (5’ AAATAATGTACGGG(T/G)GAGATGCATGA 3’) & reverse 122 (5’ GGTTCGATTGGGGTTGGTGAATATA 3’)
Primers:121 (5’ AAATAATGTACGGG(T/G)GAGATGCATGA 3’) & reverse 122 (5’ GGTTCGATTGGGGTTGGTGAATATA 3’)
Primers:121 (5’ AAATAATGTACGGG(T/G)GAGATGCATGA 3’) & reverse 122 (5’ GGTTCGATTGGGGTTGGTGAATATA 3’)
Primers:121 (5’ AAATAATGTACGGG(T/G)GAGATGCATGA 3’) & reverse 122 (5’ GGTTCGATTGGGGTTGGTGAATATA 3’)
Primers:121 (5’ AAATAATGTACGGG(T/G)GAGATGCATGA 3’) & reverse 122 (5’ GGTTCGATTGGGGTTGGTGAATATA 3’)
Primers:121 (5’ AAATAATGTACGGG(T/G)GAGATGCATGA 3’) & reverse 122 (5’ GGTTCGATTGGGGTTGGTGAATATA 3’)
Primers:121 (5’ AAATAATGTACGGG(T/G)GAGATGCATGA 3’) & reverse 122 (5’ GGTTCGATTGGGGTTGGTGAATATA 3’)
Primers:121 (5’ AAATAATGTACGGG(T/G)GAGATGCATGA 3’) & reverse 122 (5’ GGTTCGATTGGGGTTGGTGAATATA 3’)
Primers:121 (5’ AAATAATGTACGGG(T/G)GAGATGCATGA 3’) & reverse 122 (5’ GGTTCGATTGGGGTTGGTGAATATA 3’)
Primers:121 (5’ AAATAATGTACGGG(T/G)GAGATGCATGA 3’) & reverse 122 (5’ GGTTCGATTGGGGTTGGTGAATATA 3’)
Primers:121 (5’ AAATAATGTACGGG(T/G)GAGATGCATGA 3’) & reverse 122 (5’ GGTTCGATTGGGGTTGGTGAATATA 3’)
Primers:121 (5’ AAATAATGTACGGG(T/G)GAGATGCATGA 3’) & reverse 122 (5’ GGTTCGATTGGGGTTGGTGAATATA 3’)

Primers:121 (5' AAATAATGTACGGG(T/G)GAGATGCATGA 3') & reverse 122 (5' GGTTCGATTGGGGTTGGTGAATATA 3')
Primers:121 (5' AAATAATGTACGGG(T/G)GAGATGCATGA 3') & reverse 122 (5' GGTTCGATTGGGGTTGGTGAATATA 3')
Primers:121 (5' AAATAATGTACGGG(T/G)GAGATGCATGA 3') & reverse 122 (5' GGTTCGATTGGGGTTGGTGAATATA 3')
Primers:121 (5' AAATAATGTACGGG(T/G)GAGATGCATGA 3') & reverse 122 (5' GGTTCGATTGGGGTTGGTGAATATA 3')
Primers:121 (5' AAATAATGTACGGG(T/G)GAGATGCATGA 3') & reverse 122 (5' GGTTCGATTGGGGTTGGTGAATATA 3')
Primers:121 (5' AAATAATGTACGGG(T/G)GAGATGCATGA 3') & reverse 122 (5' GGTTCGATTGGGGTTGGTGAATATA 3')
Primers:121 (5' AAATAATGTACGGG(T/G)GAGATGCATGA 3') & reverse 122 (5' GGTTCGATTGGGGTTGGTGAATATA 3')
Primers:121 (5' AAATAATGTACGGG(T/G)GAGATGCATGA 3') & reverse 122 (5' GGTTCGATTGGGGTTGGTGAATATA 3')
Primers:121 (5' AAATAATGTACGGG(T/G)GAGATGCATGA 3') & reverse 122 (5' GGTTCGATTGGGGTTGGTGAATATA 3')
Primers:121 (5' AAATAATGTACGGG(T/G)GAGATGCATGA 3') & reverse 122 (5' GGTTCGATTGGGGTTGGTGAATATA 3')
Primers:121 (5' AAATAATGTACGGG(T/G)GAGATGCATGA 3') & reverse 122 (5' GGTTCGATTGGGGTTGGTGAATATA 3')
Primers:121 (5' AAATAATGTACGGG(T/G)GAGATGCATGA 3') & reverse 122 (5' GGTTCGATTGGGGTTGGTGAATATA 3')
Primers:121 (5' AAATAATGTACGGG(T/G)GAGATGCATGA 3') & reverse 122 (5' GGTTCGATTGGGGTTGGTGAATATA 3')
Primers:121 (5' AAATAATGTACGGG(T/G)GAGATGCATGA 3') & reverse 122 (5' GGTTCGATTGGGGTTGGTGAATATA 3')
Primers:121 (5' AAATAATGTACGGG(T/G)GAGATGCATGA 3') & reverse 122 (5' GGTTCGATTGGGGTTGGTGAATATA 3')
Primers:121 (5' AAATAATGTACGGG(T/G)GAGATGCATGA 3') & reverse 122 (5' GGTTCGATTGGGGTTGGTGAATATA 3')
Primers:121 (5' AAATAATGTACGGG(T/G)GAGATGCATGA 3') & reverse 122 (5' GGTTCGATTGGGGTTGGTGAATATA 3')
Primers:121 (5' AAATAATGTACGGG(T/G)GAGATGCATGA 3') & reverse 122 (5' GGTTCGATTGGGGTTGGTGAATATA 3')
Primers:121 (5' AAATAATGTACGGG(T/G)GAGATGCATGA 3') & reverse 122 (5' GGTTCGATTGGGGTTGGTGAATATA 3')
Primers:121 (5' AAATAATGTACGGG(T/G)GAGATGCATGA 3') & reverse 122 (5' GGTTCGATTGGGGTTGGTGAATATA 3')
Primers:121 (5' AAATAATGTACGGG(T/G)GAGATGCATGA 3') & reverse 122 (5' GGTTCGATTGGGGTTGGTGAATATA 3')
Primers:121 (5' AAATAATGTACGGG(T/G)GAGATGCATGA 3') & reverse 122 (5' GGTTCGATTGGGGTTGGTGAATATA 3')
Primers:121 (5' AAATAATGTACGGG(T/G)GAGATGCATGA 3') & reverse 122 (5' GGTTCGATTGGGGTTGGTGAATATA 3')
Primers:121 (5' AAATAATGTACGGG(T/G)GAGATGCATGA 3') & reverse 122 (5' GGTTCGATTGGGGTTGGTGAATATA 3')
Primers:121 (5' AAATAATGTACGGG(T/G)GAGATGCATGA 3') & reverse 122 (5' GGTTCGATTGGGGTTGGTGAATATA 3')
Primers:121 (5' AAATAATGTACGGG(T/G)GAGATGCATGA 3') & reverse 122 (5' GGTTCGATTGGGGTTGGTGAATATA 3')
Primers:121 (5' AAATAATGTACGGG(T/G)GAGATGCATGA 3') & reverse 122 (5' GGTTCGATTGGGGTTGGTGAATATA 3')
Primers:121 (5' AAATAATGTACGGG(T/G)GAGATGCATGA 3') & reverse 122 (5' GGTTCGATTGGGGTTGGTGAATATA 3')
Primers:121 (5' AAATAATGTACGGG(T/G)GAGATGCATGA 3') & reverse 122 (5' GGTTCGATTGGGGTTGGTGAATATA 3')
Primers:121 (5' AAATAATGTACGGG(T/G)GAGATGCATGA 3') & reverse 122 (5' GGTTCGATTGGGGTTGGTGAATATA 3')
Primers:121 (5' AAATAATGTACGGG(T/G)GAGATGCATGA 3') & reverse 122 (5' GGTTCGATTGGGGTTGGTGAATATA 3')
Primers:121 (5' AAATAATGTACGGG(T/G)GAGATGCATGA 3') & reverse 122 (5' GGTTCGATTGGGGTTGGTGAATATA 3')
Primers:121 (5' AAATAATGTACGGG(T/G)GAGATGCATGA 3') & reverse 122 (5' GGTTCGATTGGGGTTGGTGAATATA 3')
Primers:121 (5' AAATAATGTACGGG(T/G)GAGATGCATGA 3') & reverse 122 (5' GGTTCGATTGGGGTTGGTGAATATA 3')

Primers:121 (5’ AAATAATGTACGGG(T/G)GAGATGCATGA 3’) & reverse 122 (5’ GGTTCGATTGGGGTTGGTGAATATA 3’)
Primers:121 (5’ AAATAATGTACGGG(T/G)GAGATGCATGA 3’) & reverse 122 (5’ GGTTCGATTGGGGTTGGTGAATATA 3’)
Primers:121 (5’ AAATAATGTACGGG(T/G)GAGATGCATGA 3’) & reverse 122 (5’ GGTTCGATTGGGGTTGGTGAATATA 3’)
Primers:121 (5’ AAATAATGTACGGG(T/G)GAGATGCATGA 3’) & reverse 122 (5’ GGTTCGATTGGGGTTGGTGAATATA 3’)
Primers:121 (5’ AAATAATGTACGGG(T/G)GAGATGCATGA 3’) & reverse 122 (5’ GGTTCGATTGGGGTTGGTGAATATA 3’)
Primers:121 (5’ AAATAATGTACGGG(T/G)GAGATGCATGA 3’) & reverse 122 (5’ GGTTCGATTGGGGTTGGTGAATATA 3’)
Primers:121 (5’ AAATAATGTACGGG(T/G)GAGATGCATGA 3’) & reverse 122 (5’ GGTTCGATTGGGGTTGGTGAATATA 3’)
Primers:121 (5’ AAATAATGTACGGG(T/G)GAGATGCATGA 3’) & reverse 122 (5’ GGTTCGATTGGGGTTGGTGAATATA 3’)
Primers:121 (5’ AAATAATGTACGGG(T/G)GAGATGCATGA 3’) & reverse 122 (5’ GGTTCGATTGGGGTTGGTGAATATA 3’)
Primers:121 (5’ AAATAATGTACGGG(T/G)GAGATGCATGA 3’) & reverse 122 (5’ GGTTCGATTGGGGTTGGTGAATATA 3’)
Primers:121 (5’ AAATAATGTACGGG(T/G)GAGATGCATGA 3’) & reverse 122 (5’ GGTTCGATTGGGGTTGGTGAATATA 3’)
Primers:121 (5’ AAATAATGTACGGG(T/G)GAGATGCATGA 3’) & reverse 122 (5’ GGTTCGATTGGGGTTGGTGAATATA 3’)
Primers:121 (5’ AAATAATGTACGGG(T/G)GAGATGCATGA 3’) & reverse 122 (5’ GGTTCGATTGGGGTTGGTGAATATA 3’)
Primers:121 (5’ AAATAATGTACGGG(T/G)GAGATGCATGA 3’) & reverse 122 (5’ GGTTCGATTGGGGTTGGTGAATATA 3’)
Primers:121 (5’ AAATAATGTACGGG(T/G)GAGATGCATGA 3’) & reverse 122 (5’ GGTTCGATTGGGGTTGGTGAATATA 3’)
Primers:121 (5’ AAATAATGTACGGG(T/G)GAGATGCATGA 3’) & reverse 122 (5’ GGTTCGATTGGGGTTGGTGAATATA 3’)
Primers:121 (5’ AAATAATGTACGGG(T/G)GAGATGCATGA 3’) & reverse 122 (5’ GGTTCGATTGGGGTTGGTGAATATA 3’)
Primers:121 (5’ AAATAATGTACGGG(T/G)GAGATGCATGA 3’) & reverse 122 (5’ GGTTCGATTGGGGTTGGTGAATATA 3’)
Primers:121 (5’ AAATAATGTACGGG(T/G)GAGATGCATGA 3’) & reverse 122 (5’ GGTTCGATTGGGGTTGGTGAATATA 3’)
Primers:121 (5’ AAATAATGTACGGG(T/G)GAGATGCATGA 3’) & reverse 122 (5’ GGTTCGATTGGGGTTGGTGAATATA 3’)
Primers:121 (5’ AAATAATGTACGGG(T/G)GAGATGCATGA 3’) & reverse 122 (5’ GGTTCGATTGGGGTTGGTGAATATA 3’)
Primers:121 (5’ AAATAATGTACGGG(T/G)GAGATGCATGA 3’) & reverse 122 (5’ GGTTCGATTGGGGTTGGTGAATATA 3’)
Primers:121 (5’ AAATAATGTACGGG(T/G)GAGATGCATGA 3’) & reverse 122 (5’ GGTTCGATTGGGGTTGGTGAATATA 3’)
Primers:121 (5’ AAATAATGTACGGG(T/G)GAGATGCATGA 3’) & reverse 122 (5’ GGTTCGATTGGGGTTGGTGAATATA 3’)
Primers:121 (5’ AAATAATGTACGGG(T/G)GAGATGCATGA 3’) & reverse 122 (5’ GGTTCGATTGGGGTTGGTGAATATA 3’)
Primers:121 (5’ AAATAATGTACGGG(T/G)GAGATGCATGA 3’) & reverse 122 (5’ GGTTCGATTGGGGTTGGTGAATATA 3’)
Primers:121 (5’ AAATAATGTACGGG(T/G)GAGATGCATGA 3’) & reverse 122 (5’ GGTTCGATTGGGGTTGGTGAATATA 3’)
Primers:121 (5’ AAATAATGTACGGG(T/G)GAGATGCATGA 3’) & reverse 122 (5’ GGTTCGATTGGGGTTGGTGAATATA 3’)
Primers:121 (5’ AAATAATGTACGGG(T/G)GAGATGCATGA 3’) & reverse 122 (5’ GGTTCGATTGGGGTTGGTGAATATA 3’)
Primers:121 (5’ AAATAATGTACGGG(T/G)GAGATGCATGA 3’) & reverse 122 (5’ GGTTCGATTGGGGTTGGTGAATATA 3’)
Primers:121 (5’ AAATAATGTACGGG(T/G)GAGATGCATGA 3’) & reverse 122 (5’ GGTTCGATTGGGGTTGGTGAATATA 3’)
Primers:121 (5’ AAATAATGTACGGG(T/G)GAGATGCATGA 3’) & reverse 122 (5’ GGTTCGATTGGGGTTGGTGAATATA 3’)
Primers:121 (5’ AAATAATGTACGGG(T/G)GAGATGCATGA 3’) & reverse 122 (5’ GGTTCGATTGGGGTTGGTGAATATA 3’)
Primers:121 (5’ AAATAATGTACGGG(T/G)GAGATGCATGA 3’) & reverse 122 (5’ GGTTCGATTGGGGTTGGTGAATATA 3’)

Primers:121 (5’ AAATAATGTACGGG(T/G)GAGATGCATGA 3’) & reverse 122 (5’ GGTTCGATTGGGGTTGGTGAATATA 3’)
Primers:121 (5’ AAATAATGTACGGG(T/G)GAGATGCATGA 3’) & reverse 122 (5’ GGTTCGATTGGGGTTGGTGAATATA 3’)
Primers:121 (5’ AAATAATGTACGGG(T/G)GAGATGCATGA 3’) & reverse 122 (5’ GGTTCGATTGGGGTTGGTGAATATA 3’)
Primers:121 (5’ AAATAATGTACGGG(T/G)GAGATGCATGA 3’) & reverse 122 (5’ GGTTCGATTGGGGTTGGTGAATATA 3’)
Primers:121 (5’ AAATAATGTACGGG(T/G)GAGATGCATGA 3’) & reverse 122 (5’ GGTTCGATTGGGGTTGGTGAATATA 3’)
Primers:121 (5’ AAATAATGTACGGG(T/G)GAGATGCATGA 3’) & reverse 122 (5’ GGTTCGATTGGGGTTGGTGAATATA 3’)
Primers:121 (5’ AAATAATGTACGGG(T/G)GAGATGCATGA 3’) & reverse 122 (5’ GGTTCGATTGGGGTTGGTGAATATA 3’)
Primers:121 (5’ AAATAATGTACGGG(T/G)GAGATGCATGA 3’) & reverse 122 (5’ GGTTCGATTGGGGTTGGTGAATATA 3’)
Primers:121 (5’ AAATAATGTACGGG(T/G)GAGATGCATGA 3’) & reverse 122 (5’ GGTTCGATTGGGGTTGGTGAATATA 3’)
Primers:121 (5’ AAATAATGTACGGG(T/G)GAGATGCATGA 3’) & reverse 122 (5’ GGTTCGATTGGGGTTGGTGAATATA 3’)
Primers:121 (5’ AAATAATGTACGGG(T/G)GAGATGCATGA 3’) & reverse 122 (5’ GGTTCGATTGGGGTTGGTGAATATA 3’)
Primers:121 (5’ AAATAATGTACGGG(T/G)GAGATGCATGA 3’) & reverse 122 (5’ GGTTCGATTGGGGTTGGTGAATATA 3’)
Primers:121 (5’ AAATAATGTACGGG(T/G)GAGATGCATGA 3’) & reverse 122 (5’ GGTTCGATTGGGGTTGGTGAATATA 3’)
Primers:121 (5’ AAATAATGTACGGG(T/G)GAGATGCATGA 3’) & reverse 122 (5’ GGTTCGATTGGGGTTGGTGAATATA 3’)
Primers:121 (5’ AAATAATGTACGGG(T/G)GAGATGCATGA 3’) & reverse 122 (5’ GGTTCGATTGGGGTTGGTGAATATA 3’)
Primers:121 (5’ AAATAATGTACGGG(T/G)GAGATGCATGA 3’) & reverse 122 (5’ GGTTCGATTGGGGTTGGTGAATATA 3’)
Primers:121 (5’ AAATAATGTACGGG(T/G)GAGATGCATGA 3’) & reverse 122 (5’ GGTTCGATTGGGGTTGGTGAATATA 3’)
Primers:121 (5’ AAATAATGTACGGG(T/G)GAGATGCATGA 3’) & reverse 122 (5’ GGTTCGATTGGGGTTGGTGAATATA 3’)
Primers:121 (5’ AAATAATGTACGGG(T/G)GAGATGCATGA 3’) & reverse 122 (5’ GGTTCGATTGGGGTTGGTGAATATA 3’)
Primers:121 (5’ AAATAATGTACGGG(T/G)GAGATGCATGA 3’) & reverse 122 (5’ GGTTCGATTGGGGTTGGTGAATATA 3’)
Primers:121 (5’ AAATAATGTACGGG(T/G)GAGATGCATGA 3’) & reverse 122 (5’ GGTTCGATTGGGGTTGGTGAATATA 3’)
SL-IR- Primers: TcIII & UTCC (Burgos al al., 2007)
SL-IR- Primers: TcIII & UTCC (Burgos al al., 2007)
SL-IR- Primers: TcIII & UTCC (Burgos al al., 2007)
SL-IR- Primers: TcIII & UTCC (Burgos al al., 2007)
SL-IR- Primers: TcIII & UTCC (Burgos al al., 2007)
SL-IR- Primers: TcIII & UTCC (Burgos al al., 2007)
SL-IR- Primers: TcIII & UTCC (Burgos al al., 2007)
SL-IR- Primers: TcIII & UTCC (Burgos al al., 2007)
SL-IR- Primers: TcIII & UTCC (Burgos al al., 2007)
SL-IR- Primers: TcIII & UTCC (Burgos al al., 2007)
SL-IR- Primers: TcIII & UTCC (Burgos al al., 2007)
SL-IR- Primers: TcIII & UTCC (Burgos al al., 2007)
SL-IR- Primers: TcIII & UTCC (Burgos al al., 2007)

SL-IR- Primers: TcIII & UTCC (Burgos al al., 2007)
SL-IR- Primers: TcIII & UTCC (Burgos al al., 2007)
SL-IR- Primers: TcIII & UTCC (Burgos al al., 2007)
SL-IR- Primers: TcIII & UTCC (Burgos al al., 2007)
SL-IR- Primers: TcIII & UTCC (Burgos al al., 2007)
SL-IR- Primers: TcIII & UTCC (Burgos al al., 2007)
SL-IR- Primers: TcIII & UTCC (Burgos al al., 2007)
SL-IR- Primers: TcIII & UTCC (Burgos al al., 2007)
SL-IR- Primers: TcIII & UTCC (Burgos al al., 2007)
SL-IR- Primers: TcIII & UTCC (Burgos al al., 2007)
SL-IR- Primers: TcIII & UTCC (Burgos al al., 2007)
SL-IR- Primers: TcIII & UTCC (Burgos al al., 2007)
SL-IR- Primers: TcIII & UTCC (Burgos al al., 2007)
SL-IR- Primers: TcIII & UTCC (Burgos al al., 2007)
SL-IR- Primers: TcIII & UTCC (Burgos al al., 2007)
SL-IR- Primers: TcIII & UTCC (Burgos al al., 2007)
SL-IR- Primers: TcIII & UTCC (Burgos al al., 2007)
SL-IR- Primers: TcIII & UTCC (Burgos al al., 2007)
SL-IR- Primers: TcIII & UTCC (Burgos al al., 2007)
SL-IR- Primers: TcIII & UTCC (Burgos al al., 2007)
SL-IR- Primers: TcIII & UTCC (Burgos al al., 2007)
SL-IR- Primers: TcIII & UTCC (Burgos al al., 2007)
SL-IR- Primers: TcIII & UTCC (Burgos al al., 2007)
SL-IR- Primers: TcIII & UTCC (Burgos al al., 2007)
SL-IR- Primers: TcIII & UTCC (Burgos al al., 2007)
SL-IR- Primers: TcIII & UTCC (Burgos al al., 2007)
Sturm et al. 1989 & Wincker et al. 1994.
Sturm et al. 1989 & Wincker et al. 1994.
Sturm et al. 1989 & Wincker et al. 1994.
Sturm et al. 1989 & Wincker et al. 1994.

Host	Transmission Cycle	City of origin
Euphractus sexcinctus	Sylvatic	Caraúbas
Galea spixii	Sylvatic	Caraúbas
Human	Intradomicile	Caicó
Human	Intradomicile	Caraúbas
Human	Intradomicile	Severiano Melo
Panstrongylus lutzi	Sylvatic	Serra Negra do Norte
Panstrongylus lutzi	Sylvatic	Serra Negra do Norte
Triatoma brasiliensis	Peridomicile	Caicó
Triatoma brasiliensis	Peridomicile	Caicó
Triatoma brasiliensis	Peridomicile	Caicó
Triatoma brasiliensis	Peridomicile	Caicó
Triatoma brasiliensis	Peridomicile	Caicó
Triatoma brasiliensis	Sylvatic	Caicó
Ovis aries	Peridomicile	João Câmara
Human	Intradomicile	Serra Negra do Norte
Triatoma brasiliensis	Sylvatic	Caicó
Triatoma brasiliensis	Sylvatic	Serra Negra do Norte
Triatoma brasiliensis	Sylvatic	Serra Negra do Norte
Triatoma brasiliensis	Sylvatic	Serra Negra do Norte
Triatoma brasiliensis	Sylvatic	Serra Negra do Norte
Triatoma brasiliensis	Sylvatic	Serra Negra do Norte
Human	Intradomicile	Serra Negra do Norte
Triatoma brasiliensis	Peridomicile	Marcelino Vieira
Triatoma brasiliensis	Peridomicile	Marcelino Vieira
Panstrongylus lutzi		Serra Negra do Norte
Human	Intradomicile	Serra Negra do Norte
Triatoma brasiliensis	Peridomicile	Marcelino Vieira
Human	Intradomicile	Caraúbas

Panstrongylus lutzi	Sylvatic	Serra Negra do Norte
Triatoma brasiliensis	Peridomestic	Caicó
Triatoma brasiliensis	Sylvatic	Serra Negra do Norte
Panstrongylus lutzi	Peridomestic	Caraúbas
Panstrongylus lutzi	Peridomestic	Caraúbas
Panstrongylus lutzi	Sylvatic	Serra Negra do Norte
Panstrongylus lutzi	Sylvatic	Serra Negra do Norte
Panstrongylus lutzi	Sylvatic	Serra Negra do Norte
Panstrongylus lutzi	Sylvatic	Serra Negra do Norte
Human	Intradomicile	Caraúbas
Human	Intradomicile	Caraúbas
Human	Intradomicile	Caraúbas
Human	Intradomicile	Caraúbas
Human	Intradomicile	Caraúbas
Human	Intradomicile	Caraúbas
Human	Intradomicile	Caraúbas
Human	Intradomicile	Caraúbas
Human	Intradomicile	Caraúbas
Human	Intradomicile	Caraúbas
Human	Intradomicile	Caraúbas
Human	Intradomicile	Serra Negra do Norte
Human	Intradomicile	Caraúbas
Human	Intradomicile	Caraúbas
Triatoma brasiliensis	Sylvatic	Caicó
Triatoma brasiliensis	Sylvatic	Caicó
Triatoma brasiliensis	Sylvatic	Caicó
Human	Intradomicile	Caraúbas
Panstrongylus lutzi		Serra Negra do Norte
Human	Intradomicile	Caraúbas
Human	Intradomicile	Caraúbas
Human	Intradomicile	Serra Negra do Norte
Triatoma brasiliensis	Peridomicile	Caraúbas
Triatoma brasiliensis	Peridomicile	Santa Cruz
Triatoma brasiliensis	Peridomicile	Marcelino Vieira
Triatoma brasiliensis	Peridomicile	Marcelino Vieira

Triatoma brasiliensis	Peridomicile	Marcelino Vieira
Triatoma brasiliensis	Peridomicile	Marcelino Vieira
Ovis aries	Peridomicile	João Câmara
Triatoma brasiliensis	Sylvatic	Caicó
Triatoma brasiliensis	Sylvatic	Caicó
Triatoma brasiliensis	Sylvatic	Caicó
Human	Intradomicile	Assu
Human	Intradomicile	Caraúbas
Human	Intradomicile	Caicó
Human	Intradomicile	Dix Sept Rosado
Human	Intradomicile	Caicó
Human	Intradomicile	Acari
Human	Intradomicile	Serra Negra do Norte
Human	Intradomicile	Serra Negra do Norte
Human	Intradomicile	Severiano Melo
Human	Intradomicile	Caicó
Human	Intradomicile	Severiano Melo
Human	Intradomicile	Apodi
Human	Intradomicile	Caicó
Human	Intradomicile	Caraúbas
Triatoma brasiliensis	Peridomicile	Caicó
Triatoma brasiliensis	Peridomicile	Caicó
Triatoma brasiliensis	Peridomicile	Caicó
Human	Intradomicile	Serra Negra do Norte
Human	Intradomicile	Caraúbas
Human	Intradomicile	Serra Negra do Norte
Human	Intradomicile	Caraúbas
Triatoma brasiliensis	Peridomicile	Caicó
Triatoma brasiliensis	Peridomicile	Caicó
Triatoma brasiliensis	Peridomicile	Caraúbas
Panstrongylus lutzi		Serra Negra do Norte
Triatoma brasiliensis	Peridomicile	Caicó
Ovis aries	Peridomicile	João Câmara
Triatoma brasiliensis	Sylvatic	Caicó

Triatoma brasiliensis	Sylvatic	Serra Negra do Norte
Triatoma brasiliensis	Sylvatic	Serra Negra do Norte
Triatoma brasiliensis	Sylvatic	Serra Negra do Norte
Triatoma brasiliensis	Sylvatic	Serra Negra do Norte
Triatoma brasiliensis	Sylvatic	Serra Negra do Norte
Panstrongylus lutzi	Sylvatic	Serra Negra do Norte
Panstrongylus lutzi	Sylvatic	Serra Negra do Norte
Panstrongylus lutzi	Sylvatic	Serra Negra do Norte
Panstrongylus lutzi	Sylvatic	Serra Negra do Norte
Panstrongylus lutzi	Sylvatic	Serra Negra do Norte
Triatoma brasiliensis	Peridomicile	Caicó
Panstrongylus lutzi	Intradomicile	Caraúbas
Panstrongylus lutzi	Intradomicile	Caraúbas
Panstrongylus lutzi	Sylvatic	Serra Negra do Norte
Panstrongylus lutzi	Sylvatic	Serra Negra do Norte
Panstrongylus lutzi	Sylvatic	Serra Negra do Norte
Triatoma petrocchiae	Sylvatic	Currais Novos
Triatoma brasiliensis	Sylvatic	Currais Novos
Triatoma brasiliensis	Peridomicile	Currais Novos
Triatoma brasiliensis	Peridomicile	Currais Novos
Triatoma brasiliensis	Peridomicile	Currais Novos
Triatoma brasiliensis	Peridomicile	Currais Novos
Triatoma brasiliensis	Peridomicile	Currais Novos
Triatoma brasiliensis	Peridomicile	Currais Novos
Triatoma brasiliensis	Peridomicile	Currais Novos
Triatoma brasiliensis	Peridomicile	Currais Novos
Triatoma brasiliensis	Peridomicile	Currais Novos
Triatoma brasiliensis	Peridomicile	Currais Novos
Triatoma brasiliensis	Peridomicile	Currais Novos
Triatoma brasiliensis	Peridomicile	Currais Novos
Triatoma brasiliensis	Peridomicile	Currais Novos
Triatoma brasiliensis	Peridomicile	Currais Novos
Triatoma brasiliensis	Peridomicile	Currais Novos
Triatoma brasiliensis	Peridomicile	Currais Novos

Triatoma brasiliensis	Peridomicile	Currais Novos
Triatoma brasiliensis	Peridomicile	Currais Novos
Triatoma brasiliensis	Peridomicile	Currais Novos
Triatoma brasiliensis	Peridomicile	Currais Novos
Triatoma brasiliensis	Sylvatic	Currais Novos
Triatoma brasiliensis	Sylvatic	Currais Novos
Triatoma brasiliensis	Sylvatic	Currais Novos
Triatoma brasiliensis	Sylvatic	Currais Novos
Human	Intradomicile	Angicos
Human	Intradomicile	Caicó
Human	Intradomicile	Caraúbas
Euphractus sexcinctus	Sylvatic	RN
Triatoma brasiliensis	Peridomicile	Currais Novos
Triatoma brasiliensis	Peridomestic	Currais Novos
Triatoma brasiliensis	Peridomestic	Currais Novos
Triatoma brasiliensis	Peridomestic	Currais Novos
Triatoma brasiliensis	Peridomestic	Currais Novos
Triatoma brasiliensis	Peridomicile	Marcelino Vieira
Triatoma brasiliensis	Peridomicile	Marcelino Vieira
Triatoma brasiliensis	Peridomicile	Marcelino Vieira
Triatoma brasiliensis	Peridomicile	Marcelino Vieira
Triatoma brasiliensis	Peridomicile	Marcelino Vieira
Triatoma brasiliensis	Peridomicile	Marcelino Vieira
Triatoma brasiliensis	Peridomicile	Marcelino Vieira
Triatoma brasiliensis	Peridomicile	Marcelino Vieira
Triatoma brasiliensis	Peridomicile	Marcelino Vieira
Triatoma brasiliensis	Peridomicile	Marcelino Vieira
Triatoma brasiliensis	Peridomicile	Marcelino Vieira
Triatoma brasiliensis	Peridomicile	Marcelino Vieira
Triatoma brasiliensis	Peridomicile	Marcelino Vieira
Triatoma brasiliensis	Peridomicile	Marcelino Vieira
Triatoma brasiliensis	Peridomicile	Marcelino Vieira
Triatoma brasiliensis	Peridomicile	Marcelino Vieira
Triatoma brasiliensis	Peridomicile	Marcelino Vieira

Triatoma brasiliensis	Peridomicile	Marcelino Vieira
Triatoma brasiliensis	Peridomicile	Marcelino Vieira
Triatoma brasiliensis	Peridomicile	Marcelino Vieira
Triatoma brasiliensis	Peridomicile	Marcelino Vieira
Triatoma brasiliensis	Peridomicile	Marcelino Vieira
Triatoma brasiliensis	Peridomicile	Marcelino Vieira
Triatoma brasiliensis	Peridomicile	Marcelino Vieira
Triatoma brasiliensis	Peridomicile	Marcelino Vieira
Triatoma brasiliensis	Peridomicile	Marcelino Vieira
Triatoma brasiliensis	Peridomicile	Marcelino Vieira
Triatoma brasiliensis	Peridomicile	Marcelino Vieira
Triatoma brasiliensis	Peridomicile	Marcelino Vieira
Triatoma brasiliensis	Peridomicile	Marcelino Vieira
Triatoma brasiliensis	Sylvatic	Marcelino Vieira
Triatoma brasiliensis	Sylvatic	Marcelino Vieira
Triatoma brasiliensis	Sylvatic	Marcelino Vieira
Triatoma brasiliensis	Sylvatic	Marcelino Vieira
Triatoma brasiliensis	Peridomicile	Marcelino Vieira
Triatoma brasiliensis	Peridomicile	Marcelino Vieira
Triatoma brasiliensis	Peridomicile	Marcelino Vieira
Triatoma brasiliensis	Peridomicile	Marcelino Vieira
Triatoma brasiliensis	Peridomicile	Currais Novos
Triatoma brasiliensis	Peridomicile	Currais Novos
Triatoma brasiliensis	Peridomicile	Currais Novos
Triatoma brasiliensis	Peridomicile	Currais Novos
Triatoma brasiliensis	Peridomicile	Currais Novos
Triatoma brasiliensis	Peridomicile	Currais Novos
Triatoma brasiliensis	Peridomicile	Currais Novos
Triatoma brasiliensis	Peridomicile	Currais Novos
Triatoma brasiliensis	Peridomicile	Currais Novos
Triatoma brasiliensis	Peridomicile	Currais Novos
Triatoma brasiliensis	Peridomicile	Currais Novos
Triatoma brasiliensis	Peridomicile	Currais Novos
Triatoma brasiliensis	Peridomicile	Currais Novos

Triatoma brasiliensis	Peridomicile	Currais Novos
Triatoma brasiliensis	Peridomicile	Currais Novos
Triatoma brasiliensis	Peridomicile	Currais Novos
Triatoma brasiliensis	Peridomicile	Currais Novos
Triatoma brasiliensis	Sylvatic	Marcelino Vieira
Triatoma brasiliensis	Sylvatic	Marcelino Vieira
Triatoma brasiliensis	Peridomicile	Currais Novos
Triatoma brasiliensis	Sylvatic	Marcelino Vieira
Triatoma brasiliensis	Sylvatic	Marcelino Vieira
Triatoma brasiliensis	Sylvatic	Currais Novos
Triatoma brasiliensis	Sylvatic	Currais Novos
Triatoma brasiliensis	Sylvatic	Marcelino Vieira
Triatoma brasiliensis	Sylvatic	Marcelino Vieira
Triatoma brasiliensis	Sylvatic	Marcelino Vieira
Triatoma brasiliensis	Sylvatic	Marcelino Vieira
Triatoma brasiliensis	Sylvatic	Marcelino Vieira
Triatoma brasiliensis	Sylvatic	Marcelino Vieira
Triatoma brasiliensis	Sylvatic	Marcelino Vieira
Triatoma brasiliensis	Sylvatic	Marcelino Vieira
Triatoma brasiliensis	Sylvatic	Marcelino Vieira
Triatoma brasiliensis	Sylvatic	Marcelino Vieira
Triatoma brasiliensis	Sylvatic	Marcelino Vieira
Triatoma brasiliensis	Sylvatic	Marcelino Vieira
Triatoma brasiliensis	Sylvatic	Marcelino Vieira
Triatoma brasiliensis	Sylvatic	Marcelino Vieira
Triatoma brasiliensis	Sylvatic	Marcelino Vieira
Triatoma brasiliensis	Sylvatic	Marcelino Vieira
Triatoma brasiliensis	Sylvatic	Marcelino Vieira
Triatoma brasiliensis	Sylvatic	Marcelino Vieira
Triatoma brasiliensis	Sylvatic	Marcelino Vieira
Triatoma brasiliensis	Sylvatic	Marcelino Vieira
Triatoma brasiliensis	Peridomicile	Currais Novos
Triatoma brasiliensis	Peridomicile	Currais Novos
Triatoma brasiliensis	Sylvatic	Marcelino Vieira

Triatoma brasiliensis	Sylvatic	Marcelino Vieira
Triatoma brasiliensis	Sylvatic	Marcelino Vieira
Triatoma brasiliensis	Sylvatic	Marcelino Vieira
Triatoma brasiliensis	Sylvatic	Currais Novos
Triatoma brasiliensis	Sylvatic	Currais Novos
Triatoma brasiliensis	Sylvatic	Currais Novos
Triatoma brasiliensis	Sylvatic	Currais Novos
Triatoma brasiliensis	Sylvatic	Currais Novos
Triatoma brasiliensis	Peridomicile	Currais Novos
Triatoma brasiliensis	Peridomicile	Marcelino Vieira
Triatoma brasiliensis	Sylvatic	Currais Novos
Triatoma brasiliensis	Sylvatic	Currais Novos
Triatoma brasiliensis	Sylvatic	Currais Novos
Triatoma brasiliensis	Sylvatic	Currais Novos
Triatoma brasiliensis	Peridomestic	Marcelino Vieira
Triatoma brasiliensis	Peridomestic	Marcelino Vieira
Triatoma brasiliensis	Peridomestic	Marcelino Vieira
Triatoma brasiliensis	Peridomestic	Marcelino Vieira
Triatoma brasiliensis	Peridomicile	Currais Novos
Triatoma brasiliensis	Peridomicile	Marcelino Vieira
Triatoma brasiliensis	Peridomicile	Marcelino Vieira
Triatoma brasiliensis	Sylvatic	Caicó
Panstrongylus lutzi		Serra Negra do Norte
Triatoma brasiliensis	Peridomicile	Caicó
Triatoma brasiliensis	Peridomicile	Caicó
Triatoma brasiliensis	Sylvatic	Caicó
Triatoma brasiliensis	Sylvatic	Caicó
Triatoma brasiliensis	Sylvatic	Caicó
Triatoma brasiliensis	Sylvatic	Caicó
Triatoma brasiliensis	Sylvatic	Caicó
Triatoma brasiliensis	Sylvatic	Caicó
Triatoma brasiliensis	Sylvatic	Caicó
Triatoma brasiliensis	Sylvatic	Caicó
Triatoma brasiliensis	Sylvatic	Caicó

Panstrongylus lutzi	Sylvatic	Caraúbas
Triatoma brasiliensis	Intradomicile	Caraúbas
Triatoma pseudomaculata	Peridomicile	Olho d'Água do Borges
Panstrongylus lutzi	Sylvatic	Serra Negra do Norte
Triatoma brasiliensis	Intradomicile	Caraúbas
Triatoma brasiliensis	Peridomicile	Caicó
Triatoma brasiliensis	Peridomicile	João Câmara
Triatoma brasiliensis	Peridomicile	João Câmara
Triatoma brasiliensis	Peridomicile	João Câmara
Triatoma brasiliensis	Peridomicile	João Câmara
Triatoma brasiliensis	Sylvatic	Serra Negra do Norte
Triatoma brasiliensis	Peridomicile	João Câmara
Triatoma brasiliensis	Peridomicile	João Câmara
Triatoma brasiliensis	Peridomicile	Caicó
Triatoma brasiliensis	Peridomicile	Caicó
Triatoma brasiliensis	Sylvatic	Serra Negra do Norte
Triatoma brasiliensis	Sylvatic	Caicó
Ovis aries	Peridomicile	João Câmara
Triatoma brasiliensis	Peridomicile	Caicó
Human	Intradomicile	Serra Negra do Norte
Panstrongylus lutzi	Sylvatic	Serra Negra do Norte
Panstrongylus lutzi	Sylvatic	Serra Negra do Norte
Panstrongylus lutzi	Sylvatic	Serra Negra do Norte
Panstrongylus lutzi	Sylvatic	Serra Negra do Norte
Panstrongylus lutzi	Sylvatic	Serra Negra do Norte
Panstrongylus lutzi	Sylvatic	Caraúbas
Triatoma brasiliensis	Peridomicile	Marcelino Vieira
Triatoma brasiliensis	Peridomicile	Marcelino Vieira
Triatoma brasiliensis	Peridomicile	Marcelino Vieira
Triatoma brasiliensis	Peridomicile	Marcelino Vieira
Euphractus sexcinctus	Sylvatic	Rio Grande do Norte
